# Inhaled non-viral delivery systems for RNA therapeutics

**DOI:** 10.1016/j.apsb.2025.03.033

**Published:** 2025-03-19

**Authors:** Cheng Huang, Hongjian Li, Xing Duan, Peidong Zhang, Shaolong Qi, Jianshi Du, Xiangrong Song, Aiping Tong, Guocan Yu

**Affiliations:** a Key Laboratory of Bioorganic Phosphorus Chemistry & Chemical Biology, Department of Chemistry, Tsinghua University, Beijing 100084, China; b State Key Laboratory of Biotherapy and Cancer Center, Research Unit of Gene and Immunotherapy, Chinese Academy of Medical Sciences, Collaborative Innovation Center of Biotherapy, West China Hospital, Sichuan University, Chengdu 610041, China; c Institute for Immunology, School of Basic Medical Sciences, Tsinghua University, Beijing 100084, China; d Vascular Surgery Center, the Third Hospital of Jilin University, Changchun 130031, China

**Keywords:** Inhalation therapy, mRNA, Non-viral vectors, Lipid nanoparticle, Pulmonary diseases

## Abstract

RNA-based gene therapy has been widely used for various diseases, and extensive studies have proved that suitable delivery routes greatly help the development of RNA therapeutics. Identifying a safe and effective delivery system is key to realizing RNA therapeutics’ clinical translation. Inhalation is a non-invasive pulmonary delivery modality that can enhance the retention of therapeutic agents in the lungs with negligible toxicity, thereby improving patient compliance. Inhaled RNA therapeutics are increasingly becoming an area of focus for researchers; however, only several clinical trials have explored inhaled delivery of RNA for pulmonary diseases. This review presents an overview of recent advances in inhaled delivery systems for RNA therapeutics, including viral and nonviral systems, highlighting state of the art regarding inhalation in the messenger RNA (mRNA) field. We also summarize the applications of mRNA inhalants in infectious and other lung diseases. Simultaneously, the research progresses on small interfering RNAs (siRNAs), antisense oligonucleotides (ASOs), and different types of RNA are also discussed to provide new strategies for developing RNA inhalation therapy. Finally, we clarify the challenges inhaled RNA-based therapeutics face before their widespread adoption and provide insights to help advance this exciting field to the bedside.

## Graphical abstract

This review gives a systematic classification of RNA, viral and non-viral vectors, types of inhalers, inhaled delivery systems, and biomedical applications.

**Figure:**
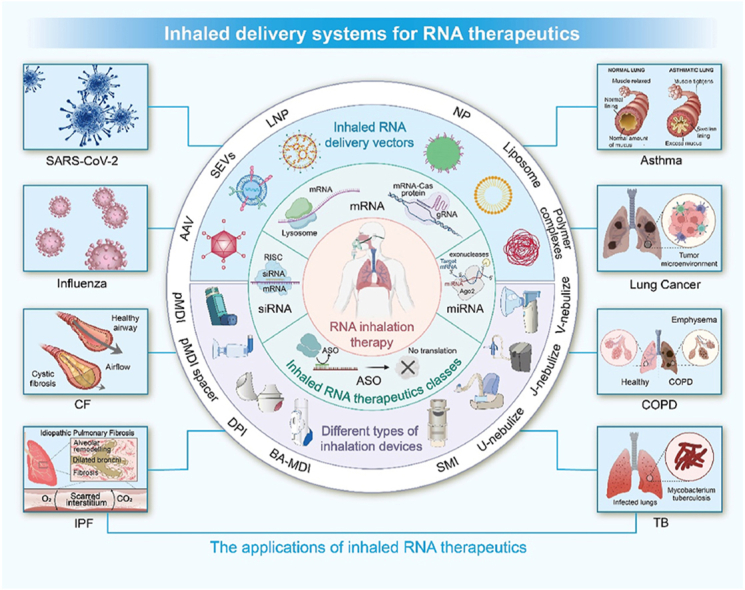

## 1 Introduction

The lung is an essential organ for respiration and gas exchange, and it is susceptible to various diseases that affect the respiratory tract, such as cystic fibrosis, asthma, and SARS-CoV-2 infection^1^^,^^2^. Among novel therapies that are currently under development for these deadly diseases, one promising strategy is the use of RNA-based therapies^3^. Combined with the current progress in inhalation research, the term ‘RNA therapeutics’ includes mRNAs, ASOs, siRNAs, and microRNAs (miRNAs). Naked RNAs injected systemically are often susceptible to degradation by serum nucleases and are rapidly cleared. As a result, viral and non-viral vectors have been applied to facilitate a more efficient delivery of RNA to cells. Viral vectors take advantage of evolutionary adaptation to encapsulate RNA and release their contents after cell binding and membrane fusion^4^. As an alternative, non-viral vectors mainly rely on natural or synthetic materials to complex or encapsulate RNA^5^. Although the efficiency of nonviral carriers still falls behind viral systems, their physical and chemical properties and surface properties can all be modified to enable more precise cell-specific targeting to reduce off-target toxicity.

The choice of administration route is crucial for developing targeted RNA therapeutics based on the anatomy and function of the lungs. Inhalation is a noninvasive pulmonary delivery route for biopharmaceuticals that offers advantages over the parenteral route, such as easy self-administration, local delivery, rapid onset of action, dose reduction, and minimized side effects^6^. Inhaled drugs can reach the entire bronchiolar and alveolar epithelium through the large surface area of the alveoli^7^^,^^8^. The advantage of inhalation, compared to intravenous or intramuscular administration routes, lies in its ability to deliver therapeutics directly to the respiratory tract, which is the targeted site of action, while requiring lower doses^9^. For example, ALN-RSV01, the first siRNA therapy targeting the RSV nucleocapsid protein, was tested clinically by pulmonary delivery using inhalation in 2008^10^. Several clinical trials on inhaled RNA therapy have been initiated. However, no inhaled mRNA therapeutics have been approved for clinical use yet due to some challenges associated with the complex pulmonary environment and the efficiency of RNA delivery. This review summarizes preclinical advances in innovative materials and delivery strategies for inhaled RNA therapeutics for treating pulmonary and other diseases. This review focuses on inhaled mRNA therapy, and the research progress for other types of RNA, such as siRNA, ASO, and miRNA, are also discussed. This review will focus on non-viral platforms used in pulmonary RNA delivery, including synthetic and biological polymers, polycomplex, liposomes, exosomes, lipid nanoparticles, and polymer-based nanoparticles, and discuss their progress and challenges. Lastly, this review presented the applications of inhaled RNAs in treating diseases, such as infectious diseases, pulmonary diseases, and lung cancer. Moreover, we point out the key issues and challenges faced by the current inhaled delivery systems, offering novel insight into the design and development of inhaled RNA therapeutics^11^^,^^12^. The clinical trials on inhaled RNA therapy are summarized in Table 1^13^, ^14^, ^15^, ^16^, ^17^, ^18^, ^19^, ^20^, ^21^, ^22^, ^23^, ^24^, ^25^, ^26^, ^27^, ^28^, ^29^, ^30^, ^31^, ^32^, ^33^. Selected recent studies with different RNA and non-viral delivery vectors that have demonstrated *in vivo* and *ex vivo* transfection in animal models are summarized in Table 2^34^, ^35^, ^36^, ^37^, ^38^, ^39^, ^40^, ^41^, ^42^, ^43^, ^44^, ^45^, ^46^, ^47^, ^48^, ^49^, ^50^, ^51^, ^52^, ^53^, ^54^, ^55^, ^56^, ^57^, ^58^, ^59^, ^60^, ^61^, ^62^.

**Table 1:** Representative inhaled RNA delivery systems in clinical trials.

Name	Indication	RNA type	Delivery system	Stage	NCT number	Ref.
ALN-RSV01	Respiratory syncytial virus infections	siRNA	N/A	Phase 2	NCT01065935	13
MIR 19®	The prevention of COVID-19	siRNA	N/A	Phase 2/3	NCT05783206	14
MBS-COV	COVID-19	siRNA	N/A	Phase 2	NCT05677893	15
siCoV/KK46	COVID-19	siRNA	Peptide dendrimer	Phase 1	NCT05208996	16
Eluforsen	Cystic fibrosis	mRNA	N/A	Phase 1/2	NCT02532764	17
SPL84	Cystic fibrosis	ASO	N/A	Phase 1/2	NCT06217952	18
MRT5005	Cystic fibrosis	CFTR-mRNA	LNP	Phase 1/2	NCT03375047	19
Aro-ENaC	Cystic fibrosis	siRNA	Ligand conjugated	Phase 1/2	NCT04375514	20
ARCT-032	Cystic fibrosis	mRNA	LNP	Phase 1	NCT05712538	21
QR-010	Cystic fibrosis	ASO	N/A	Phase 1	NCT02564354	22
ION-ENaCRx	Cystic fibrosis	ASO/Gapmer	N/A	Phase 1/2	NCT03647228	23
VX-522	Cystic fibrosis	CFTR-mRNA	LNP	Phase 1	NCT05668741	24
ExcellairTM	Asthma	siRNA	N/A	Phase 2	N/A	25
EPI-2010	Asthma	ASO	N/A	Phase 2	N/A	26
TPI ASM8	Asthma	ASO	N/A	Phase 2	NCT01158898	27
AIR645	Asthma	ASO	N/A	Phase 2	NCT00941577	28
SB-010	Asthma, COPD	ASO/DNAzyme	N/A	Phase 2	NCT01743768	29
TAKC-02	Asthma, inflammation, metabolic diseases, malignant tumors	ASO/Gapmer	N/A	Phase 1	NCT05018533	30
TPI 1100	COPD	ASO	N/A	Phase 1	NCT00914433	31
TRK-250	IPF	siRNA	N/A	Phase 1	NCT03727802	32
RCT1100	PCD	mRNA	LNP	Phase 1	NCT05737485	33

NCT numbered trials are registered at ClinicalTrials.gov; N/A: Not available.

**Table 2:** Selected studies of inhaled RNA delivery systems in animals.

Delivery vector	RNA type	Target protein	Condition	Animal model	Ref.
LNP					
7C1, phosphatidylcholine, cholesterol, PEG-lipid	mRNA	Haemagglutinin aFI6, luciferase	The H1N1 subtype of influenza A virus	BALB/c mice	34
Ionizable lipid, phosphatidylcholine, cholesterol, PEG-lipid	mRNA	__	Healthy	BALB/c mice	35,36
MC3, phosphatidylcholine, cholesterol, PEG-lipid	siRNA	Thymic stromal lymphopoietin	Asthma	BALB/c mice	37
DLin-MC3-DMA, cholesterol or *β*-sitosterol, DMG-PEG_2K_, DSPC	mRNA	CFTR	Cystic fibrosis	BALB/c mice	38
IR-117-17, DOTAP, bPEG20K, cholesterol, NaAc buffer	mRNA	Firefly luciferase	A novel nebulization delivery platform	B6, Cg-Gt (ROSA)26Sor,	39
				C57BL/6N Scnn1b-Tg	
SM102, DSPC, cholesterol, DMG-PEG, DSSC-DOPE	mRNA	SARS-CoV-2 Omicron variant and ovalbumin	SARS-CoV-2 Omicron variant and as a cancer vaccine to inhibit lung metastasis.	C57BL/6 mice	40
DPP containing mannitol and leucine	mRNA	Firefly luciferase	Lung diseases	CD-1 male mice	41
DLin-MC3-DMA, a helper lipid, cholesterol, and PEG-DMG in 5% lactose solution	siRNA	eGFP and the house keeping gene GAPDH	Respiratory diseases	An artificial mucus layer similarly found in human lungs and an adenocarcinoma cell line	42
Hybrid nanoparticles					
G0-C14, DSPE-PEG-mannose, HA	mRNA	Luciferase, green fluorescence protein, and wide-type p53	Lung cancers, pneumonia infectious diseases, and other respiratory diseases	BALB/c mice	43
PLGA-PEG and G0-C14	siRNA	Interleukin-11	Idiopathic pulmonary fibrosis	C57BL/6 mice	44
G0-C14, PLGA-PEG and [_D_-Lys6]-LHRH conjugated to PEG molecule.	siRNA	KRAS gene	Lung cancer therapy	Female nu/nu mice	45
PEI	mRNA	eGFP protein	Respiratory diseases	Mares and foals	46
Polymers					
Poly-*β*-amino-thio-ester	mRNA	Cas13a	SARS-CoV-2	BALB/c	47
				Hamster,	
				Fitch, ferrets,	
				Rhesus macaques	
Poly-*β*-amino-esters	mRNA	Cas13a	Influenza virus A and SARS-CoV-2	BALB/c mice, hamster	48
Hyperbranched poly-*β*-amino-esters	mRNA	SARS-CoV-2-neutralizing antibody	SARS-CoV-2	Hamster	8,49
Polymer complexes					
AA-PLL-PEG-MAL	mRNA	Matrix metalloproteinases	Idiopathic pulmonary fibrosis	C57BL/6J mice	50
KL4 peptide-PEG	mRNA	N/A	Healthy	BALB/c mice	51
PDMAEMA and POEGMA	siRNA	To inhibit expression of two genes [*β*III-tubulin, PLK1]	Lung cancer	C57BL/6 J mice	52
Extracellular vesicles					
HEK-exo	mRNA	IL-12	Orthotopic and metastatic lung tumors	Female C57BL/6, BALB/c and Batf3^−/−^ C57BL/6	53
Serum-derived extracellular vesicles	siRNA	Myeloid differentiation factor 88	LPS-induced lung injury	BALB/c	54
				C57BL/6 mice	
Lung-derived extracellular vesicles	mRNA	SARS-CoV-2 spike (S) protein	SARS-CoV-2	CD1 mice	55
Other					
N/A	miRNA	B[a]P-induced lung adenoma	Lung cancer prevention	A/J mice, FVB mice, and SV129 mice	56
Naked-mRNA	mRNA	Hsp65	Tuberculosis	Mice	57
ENaC-RNA	ASO	Inducing RNase H1-dependent degradation of the targeted Scnn1a mRNA.	Cystic fibrosis	Nedd4L-KO mice	58
2′-*O*-MOE phosphorothioate chemical modification-RNA	ASO	CFTR	Cystic fibrosis	Mice and monkey	59
Naked siRNA	siRNA	FAK	Hypertension	Sprague–Dawley rats	60
Chitosan/mannitol	siRNA	Luciferase	Lung metastasis	Lung metastasis	61
Nanostructured lipid carrier system	siRNA	MRP1, BCL2	Lung cancer	Athymic nu/nu mice	62

## 2 Inhalation devices and barriers

### 2.1 Compared different types of inhalers

As a noninvasive drug delivery route, inhalation relies on a well-designed inhaler. These devices can be broadly classified into pressurized metered dose (pMDI)^63^^,^^64^, breath-actuated metered-dose (BA-MDI)^65^, dry powder (DPI)^66^^,^^67^, soft mist inhalers (SMI)^68^ and nebulizers^69^. The main idea of all the devices is to obtain an aerosol of either liquid (*e.g.*, inhalers) or solid-state particles (*e.g.*, DPIs) and deliver high drug deposition in the infected area. Consistent and accurate dosing significantly impacts the effectiveness and safety of treatment. An ideal inhalation device should offer consistent performance across various usage scenarios and safeguard the medication from environmental factors like temperature and humidity. User-friendly design is crucial for ensuring proper device utilization and enhancing treatment outcomes. Furthermore, desirable attributes encompass affordability and environmentally sustainable features.

In exploratory research, nebulizers are perhaps the most accessible delivery device as they turn a liquid medicine into a fine mist that can be inhaled through the mouth or nose. There are three main types of nebulizers: jet nebulizers^70^, ultrasonic nebulizers^71^, and mesh nebulizers^72^. Mesh nebulizers utilize ultrasonic vibration film to create small and uniform droplets from liquid, forming aerosols that can be deposited effectively in the lower respiratory tract for therapeutic purposes. They are ideal for administering nanosuspensions and unstable drugs, offering an advantage over ultrasonic nebulizers by minimizing concerns related to heat-induced drug degradation. Meanwhile, Galindo-Filho et al.^72^^,^^73^ reported that mesh nebulizers delivered more radio aerosol to the lungs of COPD patients than jet nebulizers^74^ during non-invasive ventilation. From the recently published studies, mesh nebulizers are widely used in some preclinical studies of inhaled RNA delivery. Blanchard^48^, Rotolo^47^, Kim^38^ and Bai^75^ et al. used a similar principle to the mesh nebulizer to design the nebulizer for RNA delivery. It greatly improves the efficiency of RNA delivery to the lung. For inhaled drug delivery, at present, the updated review concludes that there is no significant difference in the efficacy of therapy between inhalers and nebulizers. However, it should be noted that efficiently targeted delivery vectors, formulation components, and suitable nebulizers are also important for inhaled RNA delivery. The three types of nebulizers have different working principles, performances, and applicability. Correctly choosing an appropriate nebulizer according to the patients' conditions and the drugs’ properties is crucial for treating diseases. Table 3 compares all types of inhalers^76^, ^77^, ^78^, ^79^, ^80^, ^81^. The characteristics and scope of application of the three nebulizers are summarized in Table 4^82^, ^83^, ^84^.

**Table 3:** Comparison of different inhalers.

Inhaler	Formulation	Metering system	Advantage	Limitation	Characteristic	Example	Ref.
Pressurized metered dose inhaler (pMDI) No spacer	Drug suspended or dissolved in propellant (with surfactant and cosolvent)	Metering valve and reservoir	• Patient familiarity • Portable and compact • Relatively cheap • Multidose device • Sterility and prevention of backflow • Available for most inhaled medications • Protection of the drug from light, oxygen, and water • Patient familiarity	• Requiring coordination between actuation of the device and inhalation of the dose by the patient • Contains propellants not breath-actuated • Many patients cannot use it correctly • High oropharyngeal deposition	• Co-ordination between actuation and inhalation required • Fast and deep inhalation	EasibreatheVR (Beclomethasonedipropionate salbutamol)	76, 77
pMDI with spacer	• Drug suspended or dissolved in propellant (with surfactant and cosolvent) • Spacer • Face mask (optional)	• Metering valve and reservoir • Whistle and Valve	• Easier to coordinate • Large drug doses delivered more conveniently • Less oropharyngeal deposition • Higher lung deposition than a pMDI • Slows down the aerosol cloud • Higher systemic bioavailability	• Less portable than pMDI • Require regular cleaning • Plastic spacers may acquire a static charge • The additional cost to pMDI	• Connect pMDI to spacer • Place mouthpiece between teeth and lips or place facemask over nose and mouth and form a seal	VENTOLIN™ mini spacer (salbutamol)	78, 79
Breath-actuated metered-dose inhaler BA-MDI	Drug suspended in propellant	Metering valve and reservoir	• Small size, portable, obtrusive • Quick to use • Multidose device • Breath-actuated (no coordination needed) • Cannot contaminate contents	• Contains propellants • “Cold Freon” effect Requires moderate inspiratory flow to be triggered • Drug delivery is highly dependent on good inhaler technique • Possible to get no drug in lungs with very bad technique • Most products have low lung deposition • Most products have high oropharyngeal deposition Difficult to deliver high dosses	• Breath-actuated/suspension/solution/ • Built-in dose counter • Single-handed use and manual dexterity in loading and using the device	Aptar Pharma's	80
Dry powder inhaler (DPI)	Drug blend in lactose, drug alone, drug/excipient particles	• Capsules, blisters, multidose • Blister packs reservoirs	• Handheld/portable • Breath-actuated (no coordination needed) • Does not contain propellants	• Requires a minimum inspiratory flow • May not be appropriate for emergency situations • Many patients cannot use it correctly • Most types are moisture sensitive	• Dry powder • Single handed use and manual dexterity in loading and using the device • Co-ordination between actuation and inhalation required	HandiHaler®	81
Soft mist inhaler (SMI)	Aqueous solution or suspension	Unit-dose blisters or reservoirs	• Portable and compact Multidose device • High lung deposition • The relatively long generation time of the aerosol cloud facilitates coordination of inhalation and actuation. • No propellants	• The metered volume of 15 μL limits the dose-delivery capacity of the marketed design to drugs with the adequate solubility with respect to the required dose. • The administration of tiotropium *via* RespimatVR may not be used in patients with pre-existing cardiovascular comorbidities • Not breath-actuated • Not currentl available in most countries	Co-ordination between actuation and inhalation required	RespimatVR (ipratropium bromide, olodaterol, salbutamol, tiotropium bromide)	76

**Table 4:** Comparison of different nebulizers.

Inhaler	Formulation	Metering system	Advantage	Limitation	Characteristic	Example	Ref.
Air jet nebulizer	Can nebulize solutions, suspensions, oils	Nebule dispensed into the reservoir chamber of the nebulizer	• The fuselage is large, the pilot pipe is long, and the room for movement is large • Generate aerosols from the liquid medicament using a source of compressed gas	• High amount of drug wastage • Not all drug formulations may be appropriate	Generate aerosols from the liquid medicament using a source of compressed gas	• MedixVR • Pari LC PlusVR	82
Ultrasonic nebulizer	Cannot nebulize suspensions or liquids with high viscosity/surface tension	Nebule dispensed into the reservoir chamber of the nebulizer	Large volumes of solution can be aerosolized in a relatively short time	Oscillation of the piezoelectric crystal results in the production of heat	Aerosol creation is based on the vibrations of a piezoelectric crystal that generate high frequency sound waves	• AerosonicVR • Sonix 2000 systems	83
Vibrating mesh nebulizer	Some cannot nebulize suspensions with high viscosity/surface tension	Nebule dispensed into the reservoir chamber of the nebulizer	• Portable and light • Use oscillation and a mesh membrane to induce droplet production through cavitation and wave formation in the liquid below the mesh	• Complicated and expensive • The atomization process is slightly uncomfortable • Hard to clean • Medication dosage requires adjustment • Incompatible with viscous liquids or liquids that crystallize on drying	Use oscillation and a mesh membrane to induce droplet production through cavitation and wave formation in the liquid below the mesh	• MicroAir® NE-U22 • Pari eFlow®	83,84

### 2.2 Barriers to inhalation delivery

**Figure 1:**
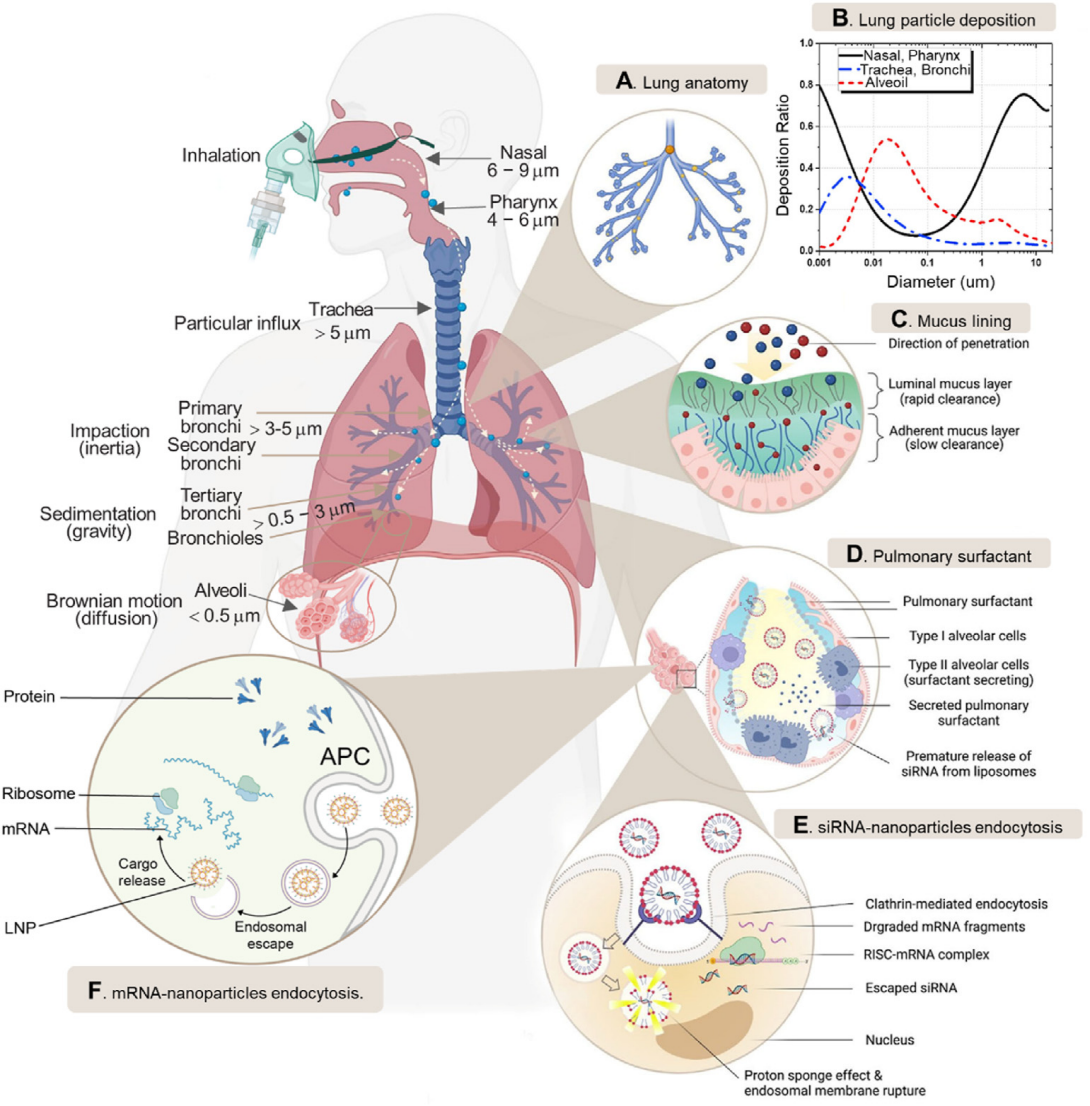
(A) Pulmonary deposition of inhaled particles and their way of separation in healthy lungs. (B) The deposition efficiency of aerosols at different regions of the respiratory tract as a function of aerosol diameter. Reprinted with the permission from Ref. 56. Copyright © 2021 Elsevier. (C,D) Hypersecreted mucus and pulmonary surfactant that could impede the therapeutic efficacy of RNA. (E) Intracellular barriers such as degradation by lysosomes must be overcome by siRNA in order for it to silence the target mRNA efficiently. Reprinted with the permission from Ref. 105. Copyright © 2023 Elsevier. (F) At the cellular level, nanocarriers should preferentially accumulate in antigen-presenting cells and efficiently release mRNA within the cytosol.

Despite the high clinical applicability of inhalation for localized RNA administration, several anatomical and physiological hurdles need to be overcome. The respiratory system possesses various defense mechanisms, including anatomical, physical, immune, and metabolic barriers^85^, ^86^, ^87^, to safeguard the gas exchange process and prevent the intrusion of external agents. Researchers are investigating strategies to enhance the stability of drug-loaded nanoparticles during inhalation, considering factors such as particle size, density, and morphology that influence lung deposition^88^. The optimal aerosol particle size for pulmonary-targeted drug release depends on the delivery system and the desired site of action^89^^,^^90^. Therefore, by tuning the size of the particles from sub-100 nm to micron-scale, one can control the location of particle deposition for pulmonary drug delivery *via* inhalation (Fig. 1A and B)^91^^,^^92^. Besides these, challenges in effective inhalation therapy include the mucus barrier^93^, diverse lung cell types hindering precise targeting^6^^,^^94^, potential components inactivation^95^, and respiratory physiology such as the air flow and the mucociliary clearance (Fig. 1C and D)^96^^,^^97^. Developing nano-formulations that efficiently penetrate the mucus layer and reach target lung cells is crucial for maximizing the efficacy and safety of inhaled therapies for lung diseases. For example, the desired therapeutic efficacy can only be achieved if target cells in the lungs take up the inhaled nanomedicine. Besides the cellular uptake, successful endosomal escape and release of siRNA or mRNA into the cytoplasm are known to be critical prerequisites that a nanocarrier must fulfill for effective siRNA delivery and the consequent knockdown or expression of specific proteins (Fig. 1E and F)^98^, ^99^, ^100^. Therefore, it is important to develop and optimize the nano**-**formulations that can efficiently penetrate the mucus layer and be absorbed by target cells in the lungs to maximize the efficacy and safety of inhaled therapy for treating lung disease. Based on understanding lung structure and barriers, inhaled medication conditions need to meet some requirements. Firstly, the aerodynamic diameter and the drug-loaded particles must meet the ideal size. Secondly, the administration of inhaled medication has to break through the mucus barrier. Thirdly, drugs like mucosa cilia and macrophages need to escape the clearance mechanism^101^^,^^102^. Targeted delivery of drugs by inhalation therapy to targeted sites could be realized though their passive targeting characteristics, active targeting characteristics, and endocytosis^103^^,^^104^. Furthermore, factors such as the patient's age, gender, the severity of illness, or the use of different types of inhalers and nebulizers are highly influential in respiratory rhythm, inspiratory flow, the volume of inspiration, and breathing break at the end of inspiration and hand-blown coordination^50^. This may be the main reason for the difficulty in inhaled RNA therapeutics in current clinical practices. Notably, with the development of mRNA modification and delivery technology, its application prospect in respiratory and pulmonary diseases is becoming increasingly widespread.

## 3 Therapeutic RNA payloads

**Figure 2:**
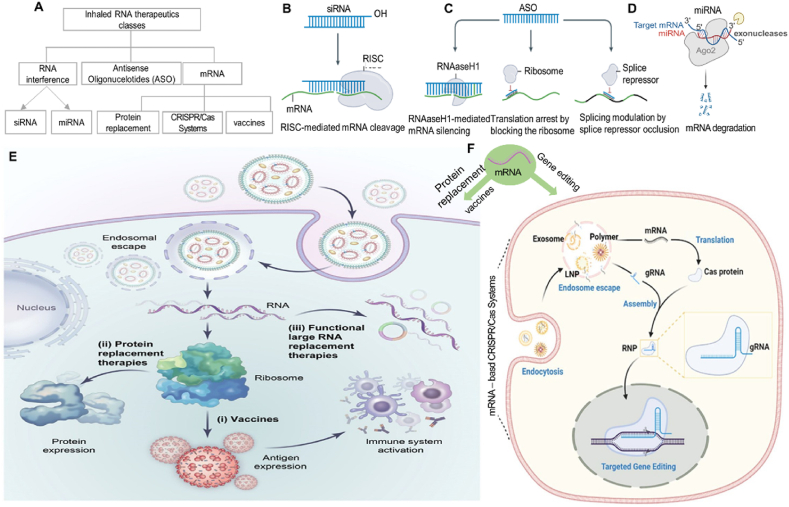
The expanding universe of therapeutic RNA payloads. (A) The diagram of inhaled RNA therapeutics classification framework. (B–D) One class of RNA therapeutics requires the delivery of small RNA molecules. siRNAs can reduce gene expression *via* RISC-mediated mRNA degradation, ASOs can alter isoforms by binding to splice sites, and miRNAs exert post-transcriptional gene regulation activity by targeting messenger RNA. Reprinted with the permission from Ref. 107. Copyright © 2022 Springer Nature. (E) Large RNAs have the potential for functional RNA replacement therapies (iii) as well as vaccines (i) or protein-replacement therapies (ii). Reprinted with the permission from Ref. 97. Copyright © 2023 Elsevier. (F) After reaching the target organs, nonviral vectors carry CRISPR/Cas mRNA and gRNA across cell membranes *via* endocytosis pathways. Following the endosomal escape, mRNA-encoded Cas proteins bind with the noncoding gRNA to form RNP. Upon entering the nucleus, RNP induces targeted gene editing. Reprinted with the permission from Ref. 108. Copyright © 2022 John Wiley and Sons.

The diverse roles of RNA in the body have led to the emergence of different approaches to harnessing RNA for therapeutic use. RNA therapeutics can be broadly divided into three functional classes: (i) inhibition of gene expression (*e.g.*, siRNA, miRNA, and ASO); (ii) protein-encoding (*e.g.*, mRNA); and (iii) protein targeting (*e.g.*, RNA aptamers)^94^^,^^106^. These RNA therapies target RNA or proteins, encode missing or defective proteins, or mediate RNA editing. Based on the diagram of the inhaled RNA therapeutics classes framework (Fig. 2A), Table 5 summarizes the characteristics of different RNA molecules for inhaled delivery and the corresponding delivery systems used for these RNAs. Oligonucleotide drugs, such as siRNAs and ASOs that utilize enzymes endogenous to eukaryotic cells, such as RNase H1 or the RNA-induced silencing complex (RISC), respectively, facilitate delivery by not requiring the delivery of large enzymes (Fig. 2B and C). One biochemical mechanism of action safely used in humans is siRNA-mediated gene silencing. These double-stranded RNAs with a molecular weight of approximately 13 kDa suppress protein translation by recruiting RISC to mRNA *via* Watson–Crick base pairing. In multiple pre-clinical studies, Bai et al.^75^ have focused on inhaled siRNA nanoparticles to improve tumor-targeting treatment of lung cancer.ASOs are a second class of RNA therapeutics oligonucleotides with a molecular weight of 6–9 kDa. ASOs have the same manufacturing advantages as siRNA and have been approved by the FDA to treat familial hypercholesterolemia^105^. In a recent study, Friedman et al.^18^ focused on SPL84, an inhaled ASO-based drug developed for treating cystic fibrosis (CF). miRNA, a small noncoding RNA, exerts post-transcriptional gene regulation activity by targeting mRNAs. Zhang and coworkers^56^ investigated the efficacy of inhaled let-7b miRNA treatment in lung cancer prevention and found that let-7b given *via* inhalation exhibited striking tumor inhibition in both the benzo[a]pyrene (B[a]P)-induced and a syngraft model of lung cancer without causing detectable side effects (Fig. 2D) mRNA therapy is a type of RNA therapeutic with several advantages over other nucleic acid therapies. It is highly efficient as it utilizes the body's natural machinery to produce proteins, resulting in higher transcription and translation rates. mRNA therapy has a favorable safety profile with short-lived molecules and limited impact on non-target tissues. It is highly adaptable and customizable for different diseases and treatment goals.

**Table 5:** Classification of inhaled RNA therapeutics.

RNA	siRNA	miRNA	ASO	mRNA
Sorting characteristic				
Structure	dsRNA	dsRNA	ssDNA or ssRNA	ssRNA
Molecular weight (kDa)	∼13	∼12–16	∼6	∼340–2300
Stability	More stable than miRNA	Unstable	Unstable	Thermally stable
				Unstable in plasma
Active site	Intracellular	Intracellular	Extracellular and intracellular	Intracellular
Mechanisms of action	mRNA decay	Complement	mRNA degradation RNA interference	mRNA translation and expression
Advantages	• Can be easily introduced into cells with high efficiency • Easily and rapidly generated • Chemical modifications possible to reduce target effects.	• Chemically synthesized • Small size • Sufficiently stable • Can be readily chemically modified • Less immunogenic than proteins	• Able to knockdown nuclear IncRNA • Easy to modify probe length to increase efficiency	• Easy to produce • Has intermediate-lasting effect • No need to enter the nucleus
Disadvantages	• Non-renewable resource • Cells might not be transfectable • Transient effect • Array format required	• Off-target effects • Not yet developed in clinical trials	• Less effective to cytoplasmic IncRNA • Higher off-target effect	• Requires special delivery methods
Inhaled delivery systems	Naked siRNA	Exosomes naked miRNA	Naked ASO	LNP
	Nanoparticles			Exosomes
	Exosomes			Nanoparticles
	Polymers			Polymers
	Liposome			

ssDNA/RNA, single-stranded DNA/RNA; dsRNA, double-stranded RNA; IncRNA, long non-coding RNA.

Additionally, mRNA therapy can activate the immune system, making it beneficial for cancer immunotherapy. The applications of mRNA therapy can be broadly categorized into prophylactic vaccines against infectious diseases, therapeutic vaccines targeting cancers, and protein-replacement therapeutics. Clinical and preclinical research has focused on using mRNA for inhaled RNA delivery (Fig. 2E and F). In terms of factors affecting inhalation delivery, apart from knowing the difference in RNA molecular weight, most RNAs are loaded *via* electrostatic binding; however, there is not currently a large number of potentially relevant studies discussing the impact of different RNA types on the efficacy of inhalation delivery. The current focus lies in the exploration of non-viral carriers. Most research discussed augmenting inhaled delivery efficiency through carrier screening and formulation optimization. Therefore, the significance of these RNA differences in the engineering of the delivery vectors and inhalation of these formulations should be further investigated.

## 4 Inhaled RNA delivery systems

Pulmonary drug delivery systems necessitate tailored design approaches that account for the nuances of administration pathways and therapeutic agents. Many specialized vectors have been engineered for the pulmonary delivery of RNA molecules, designed to enhance cellular uptake of the RNA and shield it from degradation during delivery. This section provides an exhaustive overview of the contemporary landscape of inhalation delivery vectors, encompassing exosomes, polymeric systems, polyplexes, liposomes, lipid nanoparticles, and polymer-based nanocarriers. Moreover, it delves into a thorough analysis of the most recent progress and the challenges faced in the evolution of inhalation-based RNA therapeutic delivery platforms. It underscores the critical necessity for these systems to offer augmented delivery efficiency, therapeutic effectiveness, and safety. The discussion accentuates the importance of optimizing these vectors to meet stringent requirements of respiratory drug delivery and to harness the full potential of RNA-based therapeutics in treating various pulmonary conditions.

### 4.1 Adeno-associated virus (AAV)

AAV belongs to the parvovirus family, harboring a single-stranded DNA genome of approximately 4.7 kb. The working principle of AAV vaccines involves using non-pathogenic AAV viruses for genetic delivery. The target antigen's gene sequence is inserted into the genome of the AAV virus. Once inside the body, the AAV virus releases the antigen gene sequence, enabling cells to express the antigen and induce an immune response, thereby protecting the body from infection. AAVs are widely used as gene manipulation tools in biology due to their safety, durability, high efficiency, and specificity. Chen et al.^107^ have publicly released the world's first published clinical trial results on mucosal immunity against COVID-19. The study assessed the safety and immunogenicity of the Ad5-nCoV vaccine by aerosol inhalation in adults. The research findings indicated that inhaled Ad5-nCoV was well-tolerated and did not cause any vaccine-related serious adverse events^108^. Recently, the Information Office of Shanghai Municipal People's Government announced on its official WeChat account “Shanghai Release” that the city had opened reservation registrations for booster immunizations with the CanSinoBIO recombinant COVID-19 vaccine (adenovirus type 5 vector) and stated that this inhaled COVID-19 vaccine was officially being used for immunizations. CanSinoBIO developed this adenovirus vector vaccine for COVID-19 and has been certified by the World Health Organization for emergency use^109^. Although AAV are DNA virus vector and not the primary focus of this review, the COVID-19 pandemic has prompted the proposal of utilizing inhaled delivery of AAV vaccines. The objective was to employ AAV viruses as vectors for converting COVID-19 vaccines into inhalable forms, enabling direct administration to respiratory epithelial cells. This concept offers valuable insights and translational possibilities for developing inhaled RNA therapeutics.

### 4.2 Extracellular vesicles (EVs)

**Figure 3:**
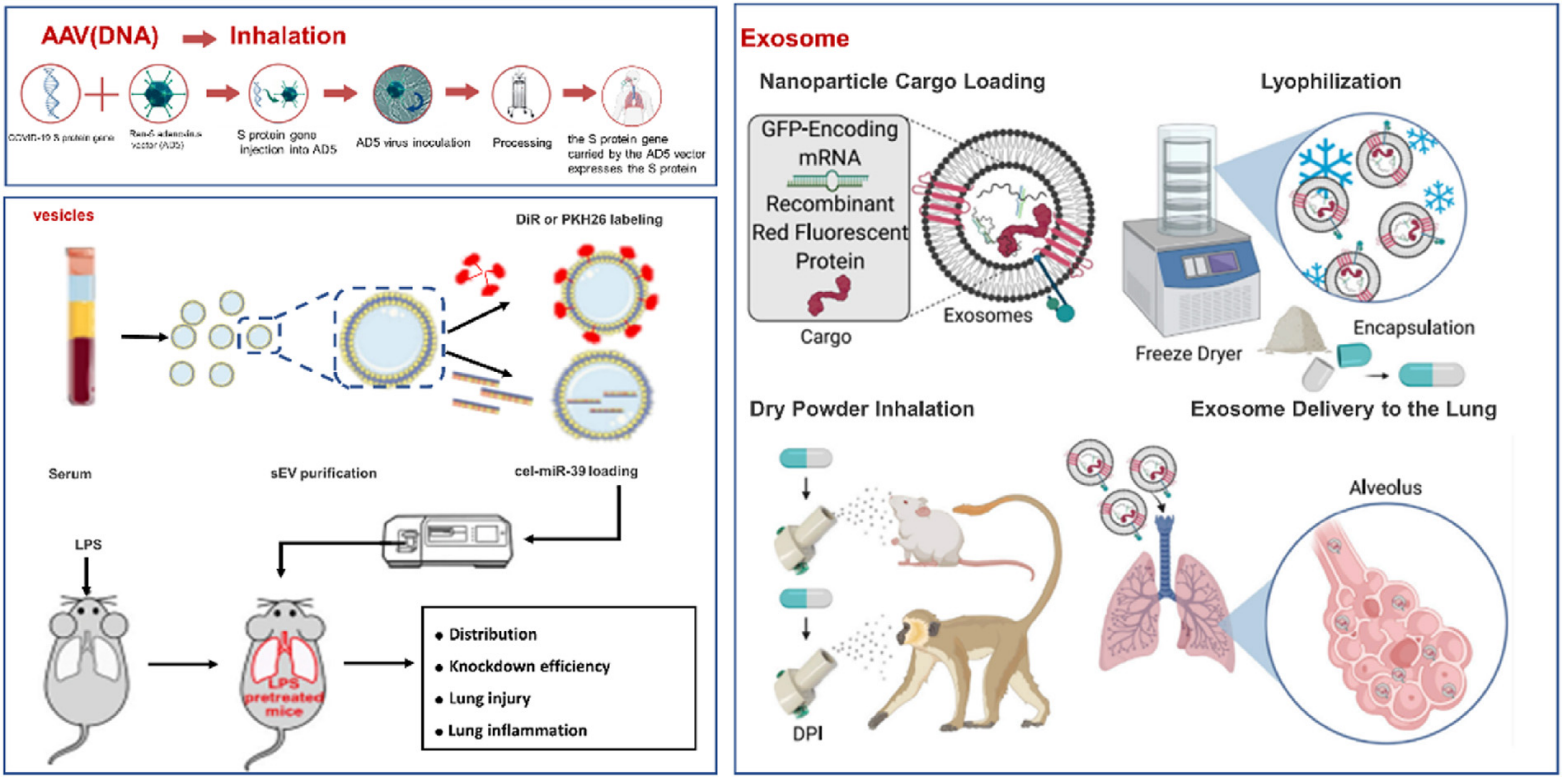
Delivery systems for nucleic acid inhalation, including AAV, extracellular vesicles, Reprinted with the permission from Ref. 54. Copyright © 2022 Elsevier. And exosomes, Reprinted with the permission from Ref. 55. Copyright © 2022 Elsevier.

EVs with suitable size, lipophilicity, and surface proteins have garnered extensive interest as delivery systems because they can package diverse contents, penetrate through cell membranes, and release their contents intracellularly. At the same time, EVs of homogeneous origin acting as delivery systems negligibly induce the innate immune responses that are always elicited by artificial delivery vectors^110^. A recent study showed that a vibrating mesh nebulizer could deliver serum-derived EVs to murine lungs. *In vivo* EV tracking revealed that inhaled EVs were distributed exclusively in the lungs and localized mainly in lung macrophages and airway epithelial cells. Furthermore, EVs loaded siRNAs through inhalation could attenuate lipopolysaccharide-induced lung injury in mice, supporting the use of this inhalation method to deliver functional small RNAs^54^. EVs come in several types, including micro-vesicles, exosome-like vesicles, exosomes, and membrane particles. There have been studies on exosomes as delivery carriers of nucleic acid drugs^111^^,^^112^. Liu et al.^53^ selected lung-derived exosomes (lung-exo) with natural lung homing ability for the inhalation delivery of mRNA. The lung-exo maintained its original function after one month of storage at room temperature, indicating high stability. This work has greatly promoted the delivery of nucleic acid therapeutics using functional exosomes as platforms for inhaled drug delivery. However, Lipo does not represent LNPs containing other lipid molecules, including ionizable, cholesterol, helper, and PEG conjugated-lipid. Therefore, it is still unclear whether exosomes have a betterinhaled delivery effect than LNP (Fig. 3)^113^.

### 4.3 Polymers

As early as 2000, Densmore et al.^114^ researched DNA inhalation delivery. Researchers compared PEI-DNA formulations and cationic lipid-DNA formulations in the inhalation system. For example, DC-cholesterol (3(-[*N*-[(*N*,*N*-dimethyl-amino)ethane]carbamoyl]cholesterol), guanidinium cholesterol (BGTC), GL-67 (N4-spermine hydrayl carbamate), 1,2-dilauroyl-*sn*-glycero-3-ethylphos-phocholine (DL-EPC), *N*-(2-hydroxyethyl)-*N*,*N*-dimethyl-2,3-bis(tetradecytoxy)-1-propanaminium bromide (DMRIE), and dioleoyl trimethylammonium propane (DOTAP) were used to prepare the cationic lipid-DNA formulations, which exhibited obvious advantages in delivery efficiency over PEI formulations. PEI produces efficient expression of delivered DNA encoding human growth hormone and produces specific antibodies that are several times higher than those of cationic lipid-DNA formulations. In another polymer-based inhalation delivery system, Kumari Patel et al.^8^ synthesized hyperbranched poly(beta amino esters) (hPBAEs) to enable nanoformulations of stable and concentrated polyplexes for inhalation. Importantly, repeat dosing of inhaled hPBAEs-mRNA generated consistent protein production in the lung without local or systemic toxicity. The efficiency of this delivery system based on hPBAEs was much better than that based on PEI. Another study on hyperbranched polymers also demonstrated their advantages in inhaled mRNA delivery. Rotolo et al. synthesized a poly-*β*-amino-thioester (PBATE) (P76), which enabled effective delivery of mRNA regardless of cargo size and complexity to hamsters, ferrets, cows, and rhesus macaques. P76 was safe and well tolerated, with greater expression than previous inhaled PBAE candidates. It allowed for fourfold dose sparing compared with previous inhaled PBAEs in a Casl3a efficacy study against SARS-CoV-2 in the Syrian hamster model. This approach was also shown to be competitive with high doses of intraperitoneal-delivered neutralizing antibodies, COV2-2381, which is a gold-standard control^115^.

Polymer materials also have a wide range of applications in the inhalation delivery of siRNA. For example, Conti et al.^64^^,^^116^ developed a siGFP–polymer conjugate that preserved silencing efficiency for up to 40% dispersed in HFA using a 4th generation dendrimer poly(amidoamine) and a mannitol or CSLA co-oligomer shell to achieve microparticle formation upon inhalation. The polyplex also showed promising aerosol performance, with 46%–49% (*w*/*w*) of inhaled siRNA reaching the lower respiratory tract to be therapeutically beneficial. McCarroll et al.^52^ developed a polymer-based star-siRNA nanoparticle. By aerosolizing the nanoparticle, the researchers observed that they could accumulate in the lungs and silence the expression of beta III-tubulin and Polo-Like Kinase 1 (PLK1) in lung tumors in mice, which delays tumor growth.

### 4.4 Polycomplex

Ternary complex containing polymer, peptide, and nucleic acid has also been used for mRNA inhalation. Qiu et al.^51^ attached cationic KL4 peptide to a monodisperse linear PEG of 12-mer to synthesize PEG_12_KL4, which formed nanosized complexes with mRNA at 10:1 ratio (*w*/*w*). *In vivo* studies demonstrated PEG_12_KL4 enhanced mRNA uptake in the lungs of BALB/c mice compared to naked mRNA or mRNA-lipoplexes, resulting in effective transfection in human lung epithelial cells. Besides, Guan et al.^117^ employed poloxamine-based copolymers, peptides, DNA, or mRNA to build a ternary complex for drug inhalation. The delivery efficiency of this ternary complex was much higher than that of the cationic lipid DOTAP. The peptides developed by modular design approaches could spontaneously form compact and monodisperse nanoparticles with poloxamine and nucleic acids *via* self-assembly. Both mRNA and plasmid DNA expression mediated by peptide-poloxamine nanoparticles is greatly boosted *in vitro* and the lungs of cystic fibrosis mice. Notably, negligible toxicity was monitored during therapy, indicating promising potential of this strategy in clinic translation. More recently, an inhaled ribosomal protein-based mRNA nanoformulation was reported to clear the intrapulmonary extracellular matrix and re-epithelialize the disrupted alveolar epithelium, thereby reversing established fibrotic foci in idiopathic pulmonary fibrosis. A ribosomal protein-condensed mRNA core, a bifunctional peptide-modified corona and keratinocyte growth factor with a PEGylated shielding shell sequentially assembled the nanoformulation. In this polymer nanoparticle, the P–N3 terminal presented on the carrier loaded nucleic acid with DBCO *via* a click reaction^50^.

### 4.5 Lipid nanoparticles (LNPs)

**Figure 4:**
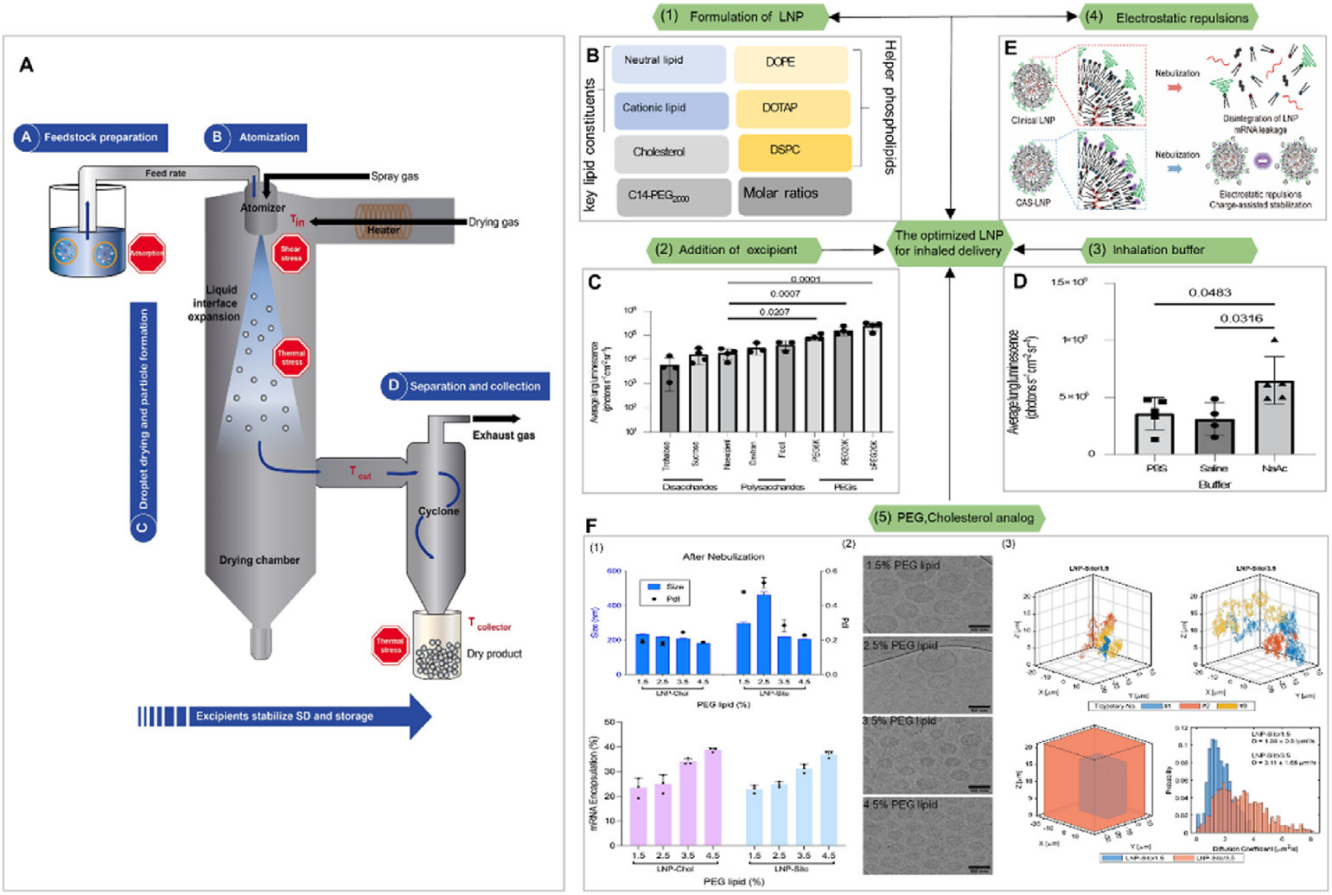
Surveying how five LNP chemical traits influence inhaled mRNA delivery. (A) The spray drying process consists of four main stages. Reprinted with the permission from Ref. 130. Copyright © 2023 Elsevier. (B) Several factors might influence the prescribing pattern of LNP, such as ionizable and cationic lipids, PEG lipids, helper phospholipids and cholesterol^34^^,^^39^. (C,D) Excipients and buffer modifications for improved LNP stability and *in vivo* delivery. (C) Bioluminescence in lungs 6 h after inhaled administration of 0.5 mg firefly luciferase mRNA with different formulations and (D) PEG excipients showing a significant increase over no excipients change in mRNA encapsulation before and after inhalation. Reprinted with the permission from Ref. 39. Copyright © 2023 Springer Nature. (E) Schematic illustrating the preparation and mechanism of CAS-LNP. Incorporating charged lipids into clinical LNP formulation, the increased electrostatic repulsions among CAS-LNPs enhanced LNP stability during inhalation. Reprinted with the permission from Ref. 40. Copyright © 2023 Chem Rxiv. This content is a preprint and has not been peer-reviewed. (F) Improved LNP inhaled effect by PEG concentration and cholesterol substitution with *β*-sitosterol in LNPs. (1) Change in mRNA encapsulation before and after nebulization. (2) CryoTEM image of LNP containing varying amounts of PEG lipids. (3) 3D-SMART results to capture nanoparticle diffusion. Reprinted with the permission from Ref. 38. Copyright © 2022 American Chemical Society.

LNP-based delivery systems are predominantly utilized for intravenous, subcutaneous, and intramuscular injections. In the case of LNPs-enabled RNA vaccines, a tightly regulated cold chain infrastructure is necessary^118^, ^119^, ^120^, ^121^, ^122^, ^123^. Parenteral formulations face further hurdles, such as the instability of liquid formulations at ambient temperatures, the need for skilled healthcare personnel, patient reluctance towards this delivery method (due to needle phobia, injection site pain, and risks of unintended local or systemic immune responses), and the potential for contamination (related to needle or injection site). These challenges have been thoroughly examined in previous studies^124^. Some of these challenges could be overcome by changing to a dry powder formulation suitable for inhalation. Friis et al.^125^ described a proof-of-concept study on engineering an mRNA-LNPs formulation suitable for spray drying. This process produced a dry powder formulation that maintained stability and preserved mRNA functionality with increased performance compared to liquid formulations stored for two weeks at 4 °C. The spray-dried LNPs may be used in future research for inhalation or intratracheal delivery systems. The process is illustrated in Fig. 4A. As current clinical formulations are not optimized for lung inhalation, effectively addressing the complex pulmonary vaccine landscape necessitates a meticulous reassessment of mRNA formulation strategies that can enhance stability and prolong shelf life. Among other methods, we summarized the most recently published studies on the use of mRNA-LNPs in inhaled delivery systems. They have made a series of optimizations to improve LNPs inhalation delivery. These were then categorized into the following five aspects: LNPs formulation, excipient addition, inhalation buffer, electrostatic repulsions, PEG concentration, and cholesterol analog. The properties of inhaled LNP, both structural and biological, can be attributed to a single factor and the optimal combination(Fig. 4B)^34^^,^^39^. Both Dahlman and Anderson analyzed prescription screening for the components of inhaled LNPs. Fortunately, Lokugamage and coworkers^34^ revealed the development strategy for inhalation therapy in their research. The employment of 7Cl lipid caused the 28-fold difference in delivery between the best- and worst-performing LNPs simply by changing the formulation ratio. In addition, the team proposed three principles for inhaled LNPs delivery systems: (1) PEG-lipids were essential for forming stable 7C1-based LNPs structures. (2) Combining cation-assisted lipids and a high molar percentage of PEG resulted in increased mRNA delivery after inhalation. (3) LNPs formulated with neutral phospholipid lipids required less PEG than cation-assisted lipids. Based on these three principles, the research team constructed an LNP called Nebulized Lung Delivery 1 (NLD1) for further analysis. By comparing the relative size of different dynamic light scattering peaks, the researchers found that NLD1 was more stable than cKK-E12 and MC3. Finally, NLD1 was stable and well tolerated after inhalation, and the lung transfection efficiency was higher. The lung intensively expressed the delivered mRNA and all six mice infected with the virus survived the therapeutic period. After the initial screening of inhaled LNPs prescription, Anderson observed that LNPs formulations could be stabilized to resist inhalation-induced aggregation by altering the inhalation buffer to increase the LNPs charge during inhalation and by the addition of a branched polymeric excipient (Fig. 4C and D)^39^. This will greatly improve the inhalation effect of mRNA-LNPs. In addition, a dense PEG layer helps LNPs achieve pulmonary delivery of mRNA following inhalation^126^^,^^127^. The density of PEG has an impact on the random motion of nanoparticles, which subsequently alters their movement within mucus^128^^,^^129^. Studies have demonstrated that an increase in PEG content leads to higher efficiency in encapsulating mRNA and a reduction in the size of LNPs. However, an excess amount of the PEG layer inhibited receptor-mediated endocytosis by decreasing the adsorption of serum proteins and hindering the escape of LNPs from endosomes, thereby significantly limiting the intracellular delivery of mRNA^130^, ^131^, ^132^. Therefore, Kim et al.^38^ exploited *β*-sitosterol, a phytosterol facilitating LNP’ endosomal escape, to meet such criteria. They adopted that combinations of *β*-sitosterol and high PEG contents would permit nebulization, mucus penetration, and endosomal escape of LNPs. This inhaled LNPs retained its physicochemical properties and efficiently delivered mRNA after inhalation (Fig. 4E). Interestingly, in a recent study, Liu et al.^40^ developed a charge-assisted stabilization (CAS) strategy aimed at inducing electrostatic repulsions among LNPs to enhance their colloidal stability. By optimizing the surface charges using a peptide–lipid conjugate, the leading CAS-LNPs demonstrated exceptional stability during inhalation (Fig. 4F). These factors affect the effectiveness and controllability of the inhaled mRNA-LNP delivery system.

In general, LNPs-based inhalation delivery systems should be the most advanced delivery systems in development. At least four inhaled mRNA nanocarriers have been used in clinical trials worldwide. MRT5005, developed by Translate Bio, an mRNA therapy research and development company in the United States, was approved to enter clinical trials for cystic fibrosis in 2018 and was the world's first LNPs-mRNA drug to enter clinical trials in an aerosol preparation^133^. Inhaled LNPs-mRNA drugs (ARCT-032, VX-522, and RCT1100) were approved for clinical trials in 2023 to treat cystic fibrosis lung disease and primary ciliary dyskinesia (PCD). Unfortunately, developing inhalation gene therapies can be more complex than other delivery methods. Despite their significant impact, inhaled gene therapies for CF have yet to enter the market due to disappointing results in human trials despite promising preclinical research.

### 4.6 Cationic liposomes

**Figure 5:**
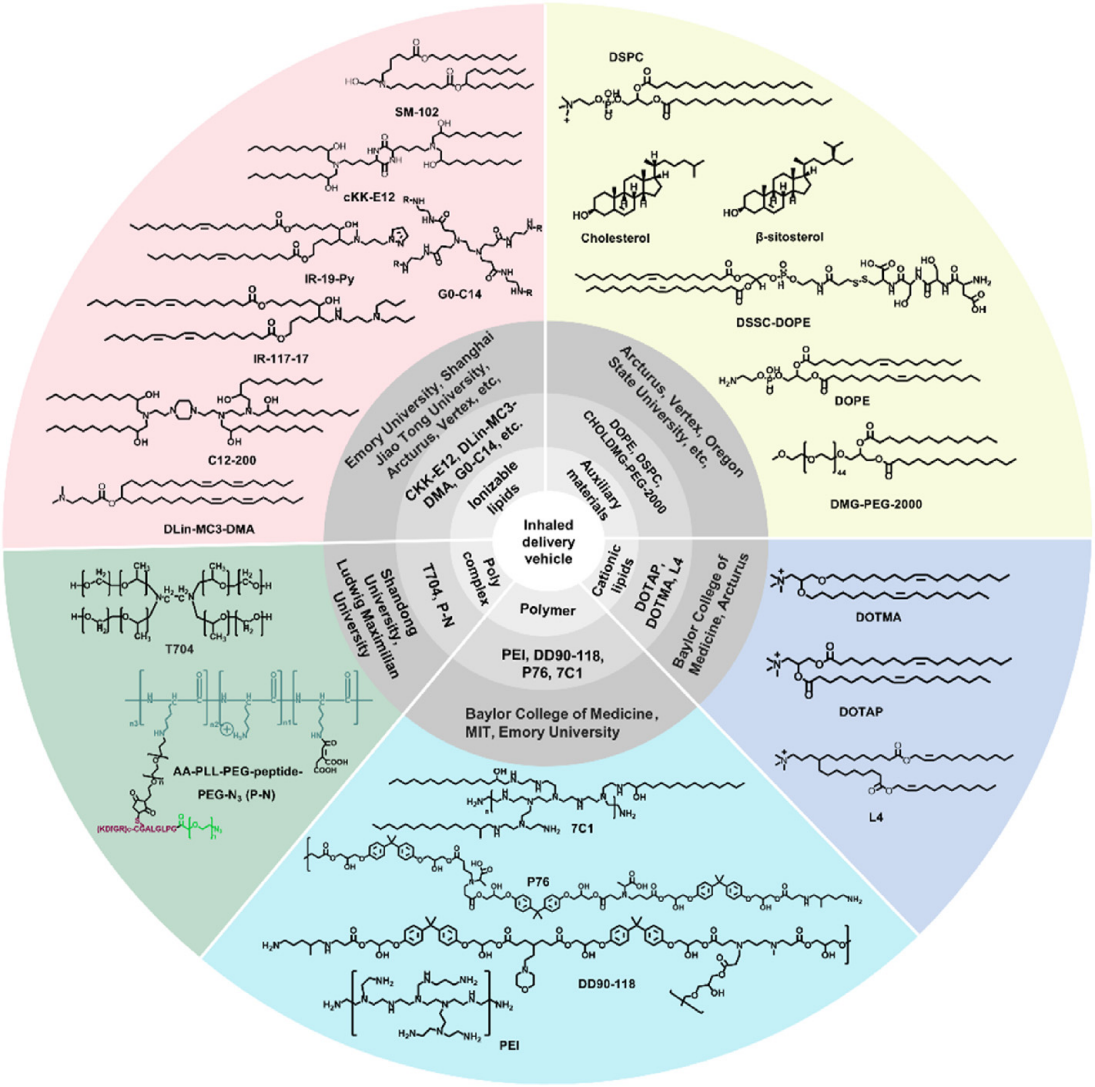
Related carriers for inhaled RNA delivery. Among nanoparticles loaded with RNA positively charged delivery carriers are the most commonly used. Positively charged vectors play a crucial role in the delivery of nucleic acid drugs because they wrap the mRNA through electrostatic adsorption between them and the negatively charged nucleic acid. These carriers can be classified in polymer, polycomplex, cationic, and ionizable lipids. Here, we listed representative polymer carriers (DD90-118, P76, 7C1), polycomplex (T704, P–N), cationic lipids (DOTAP, DOTMA, L4), ionizable lipids (CKK-E12, DLin-MC3-DMA, G0-C14, SM102, IR-117-17, IR-19-Py, C12-200). In addition, common auxiliary materials were summarized, including DOPE, DSPC, cholesterol, *β*-sitosterol, and DMG-PEG-2000. Developing an efficient delivery carrier and optimizing the prescription can be used to realize the efficient inhaled delivery of RNA.

Cationic liposomes were the earliest nucleic acid carriers and have an extensive research basis. A majority of studies have also explored inhaled delivery of cationic liposomes. DOTAP and DOTMA are the most representative ionizable lipids due to their extensive research foundation, relatively low cost, and ease of synthesis. Pei et al.^134^ synthesized functional analogs of DOTAP and successfully incorporated them into mRNA-wrapping cLNPs. These cLNPs have similar physicochemical properties to cLNPs using DOTAP, but the delivery effect was not significantly better than DOTAP. Although a large number of studies have shown that delivery systems based on ionizable lipids are significantly superior to cationic delivery systems, the use of cationic carriers as auxiliary lipids has been extensively studied and proved to have some unique advantages. From this perspective, it may provide a meaningful reference for future research. We present the related carriers for inhaled delivery of RNAs in Fig. 5.

### 4.7 Nanoparticles (NPs)

**Figure 6:**
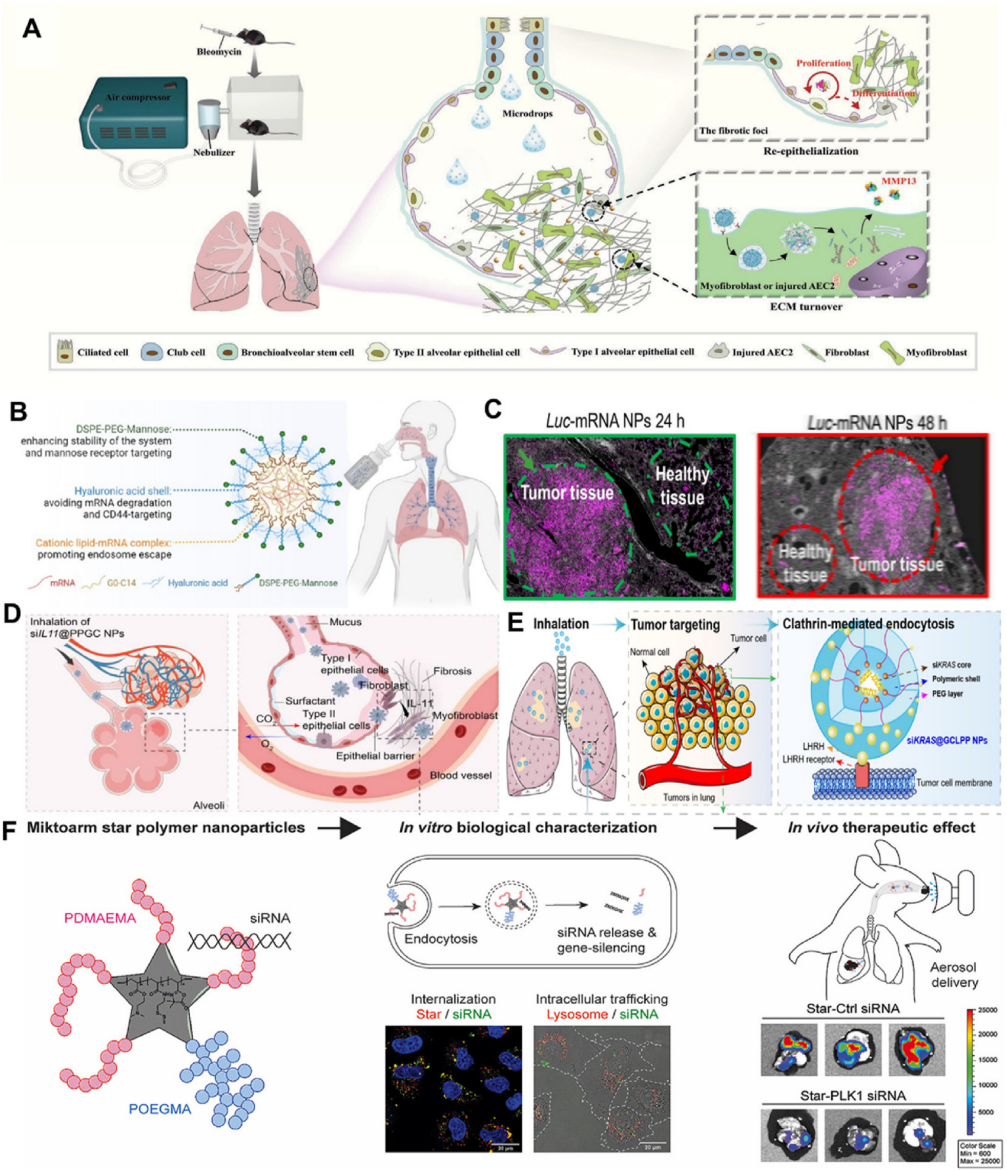
(A) Therapeutic mechanism of mMMP13@RP/P-KGF. After inhaled delivery in a mouse with IPF induced by bleomycin, the mMMP13@RP/P-KGFs deposited in the fibrotic foci were cleaved into KGF and mMMP13@RP/P by overexpressed MMP2 to achieve a synergistic antifibrosis effect based on ECM turnover and re-epithelialization. Reprinted with the permission from Ref. 50. Copyright © 2022 John Wiley and Sons. (B) Schematic illustration of the preparation and inhalation delivery of dual-targeted mRNA HDPM NPs. (C) IF staining of lung tissues from mice with orthotopic non-small cell lung cancer after inhalation of empty HDPM NPs or firefly luciferase mRNA HDPM NPs. Reprinted with the permission from Ref. 43. Copyright © 2023 National Academy of Sciences. (D) Inhaled delivery of siRNA-encapsulated PPGC NPs to MLFs for the treatment of IPF. Reprinted with the permission from Ref. 75. Copyright © 2022 The American Association for the Advancement of Science. (E) Schematic illustration of inhalable siKRAS@GCLPP NPs for suppressing KRAS-mutant NSCLC. Reprinted with the permission from Ref. 45. Copyright © 2022 American Chemical Society. (F) Miktoarm star NPs self-assemble siRNA into form small, non-toxic nanocomplexes. Reprinted with the permission from Ref. 52. Copyright © 2022 Elsevier.

The advancements in nanotechnology have facilitated the development of nanoparticle-based drug delivery systems for cancer immunotherapy^135^. Researchers have demonstrated the efficacy of utilizing nanoparticle carriers with a precise size of 100 nm to enhance drug retention duration and concentration within solid tumors through the augmented permeability and retention effect following intravenous administration^136^. However, there remains considerable scope for enhancing the efficiency of drug accumulation, specifically at targeted tumor sites^137^. Simultaneously, the intricate fabrication process and high-cost is barriers to the widespread clinical implementation of these nanoparticle drug carriers^138^. Due to the distinctive physiological state of the lungs within the respiratory system, the noninvasive administration of drugs through aerosol nebulization has demonstrated exceptional benefits in managing respiratory ailments^139^, ^140^. For mRNA, Zhang et al.^50^ sought to develop inhaled nanoparticles co-delivering the messenger RNA of MMP13 and KGF to fibrotic lung tissues to reverse established pulmonary fibrosis in a bleomycin-induced murine model. Tang et al.^43^^,^^47^ recently also reported a novel approach involving the development of dual-targeted mRNA NPs using cationic lipid and hyaluronic acid. The designed NPs exhibited excellent stability and demonstrated efficient transfection of targeted proteins into lung tissues. Importantly, the optimized dual-targeted mRNA NPs exhibited a dual capacity: They primarily accumulated in lung tumor cells and inflammatory macrophages following inhalation delivery, enabling effective expression of desired proteins (Fig. 6A–C). Regarding siRNA delivery, Zhao et al.^45^^,^^75^ prepared an inhalable and mucus-penetrative NP system incorporating siRNA against IL11 and KRAS-mutant NSCLC based on ionizable lipid compound G0-C14. This work presented a versatile NP platform for the locally inhaled delivery of siRNA therapeutics. It exhibited promising clinical potential in treating numerous respiratory diseases, including IPF and KRAS-mutant NSCLC (Fig. 6D and E). In addition, Ma et al.^52^ investigated the potential of inhaled star-siRNA NPs to accumulate in orthotopic mouse lung tumors to inhibit gene expression of *β*III-tubulin and Polo-Like Kinase 1, which were upregulated in lung cancer cells and promoted tumor growth (Fig. 6F). These results indicated a proof-of-concept for inhaled delivery of RNA nanoparticles as a novel therapeutic strategy to treat respiratory-related disease and tumor.

## 5 The applications of inhaled RNA delivery systems

### 5.1 SARS-CoV-2

#### 5.1.1 Systematic and mucosal immune responses

**Figure 7:**
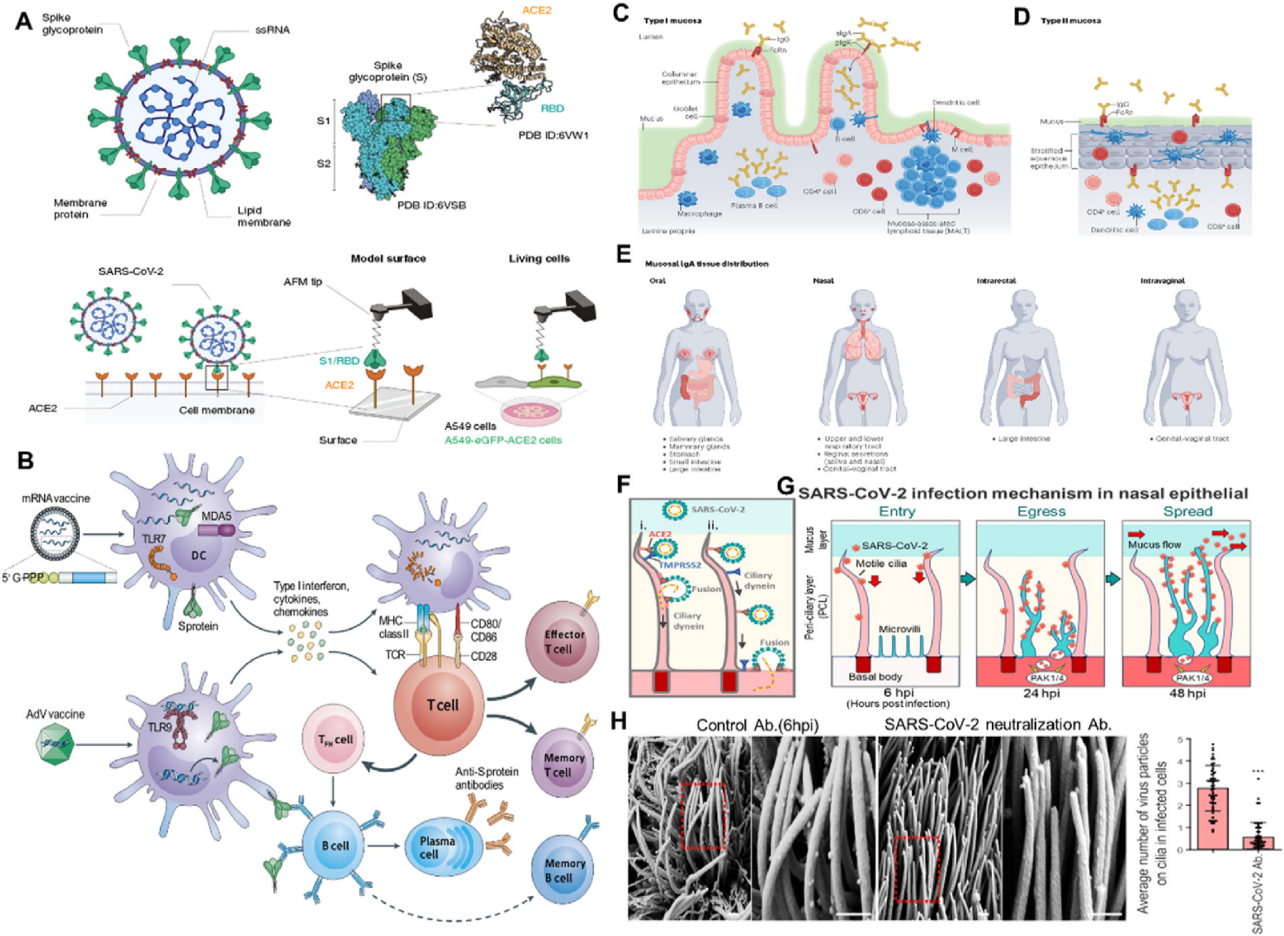
(A) SARS-CoV-2 entry into the cell. SARS-CoV-2 binds to ACE2 through spike protein's receptor-binding domain (RBD). Reprinted with the permission from Ref. 149. Copyright 2020 Spring Nature. (B) mRNA and adenovirus vector vaccines elicit immunity to SARS-CoV-2. Reprinted with the permission from Ref. 154. Copyright © 2021 Spring Nature. (C–E) Schematic of type I and II mucosal tissues and mucosal tissue IgA distribution. Reprinted with the permission from Ref. 155. Copyright © 2023 Spring Nature. (F) Model for motile cilia during SARS-CoV-2 entry. (G) Model for SARS-CoV-2 entry, egress, and spread in the nasal airway. (H) SARS-CoV-2 neutralization antibody inhibits attachment of SARS-CoV-2 to cilia. Reprinted with the permission from Ref. 152. Copyright © 2023 Elsevier.

The SARS-CoV-2 virus, responsible for the COVID-19 pandemic, continues to evolve through mutations and genetic recombination, resulting in new variants^121^. These variants pose challenges to existing management strategies and vaccine efficacy^122^. Currently, mRNA vaccines like mRNA1273 (Moderna) and BNT162b2 (Pfizer) are widely used to provide systemic immunization against SARS-CoV-2^141^, ^142^, ^143^. However, these vaccines require injection and frozen storage, making accessibility and delivery difficult, particularly in lower-income-countries. To address these challenges, there is a need to explore alternative formulation and delivery methods, such as inhalation, for mRNA vaccines, which could offer easier storage requirements and enhanced mucosal protection^144^, ^145^, ^146^. Working from the principle of SARS-CoV-2 virus infection, this virus migrates towards the posterior region of the nasal passageways, where it binds to and penetrates host cells *via* the ACE2 present on the membrane of bronchial epithelial cells (Fig. 7A)^147^, ^148^, ^149^. After entering the dendritic cells (DCs), The two vaccine formulations-LNP or adenovirus (AdV) vectors encoding the S protein, could produce high levels of S protein, which were produced by the host cell and could induce an immune response. Meanwhile, the mRNA vaccines also shortened time using the body's molecular mechanisms (Fig. 7B). However, more than 90% of pathogens enter the body through the mucosal site. The primary site of SARS-Cov-2 replication is the upper respiratory tract mucosa. More importantly, type I mucosa tends to focus on the areas of the respiratory (the upper respiratory tract (URT) and the lower respiratory tract (LRT) tract mucosa. Mucosal DCs can migrate and transport antigens to systemic inductive sites such as the lymph nodes and spleen. It has often been demonstrated that mucosal vaccination induces robust systemic humoral immunity, eliminating virus particle that evades the main immune response at the mucosal site^150^^,^^151^. Equally important, effective protection against pathogens necessitates the coordinated engagement of both the systemic and mucosal immune responses, employing the production of both immunoglobulin G (IgG) and IgA antibodies. Therefore, it is essential to examine the persistence of vaccine efficacy and the vaccine-induced mucosal immunity for SARS-CoV-2 prevention (Fig. 7C–E)^145^^,^^152^^,^^153^. Recent results showed that SARS-CoV-2 virus was delivered to the respiratory tract through inhalation and attaches first to airway multi-cilia *via* the ACE2 receptor (Fig. 7F). Normally, viruses are impenetrable to the pericililiary layer. However, they can use motile cilia as tracks to access the cell body and achieve infection of ciliated epithelial cells. Moreover, upon initial SARS-CoV-2 virus contagion, the SARS-CoV-2 hijacks the host cell machinery to induce elongated and highly branched microvilli, and this microvillus enables the virus to exit across the PCL layer before lateral spread to other regions (Fig. 7G). Importantly, an anti-spike glycoprotein monoclonal antibody that neutralizes SARS-CoV-2 inhibited attachment of SARS-CoV-2 to cilia and decreased infected cell numbers (Fig. 7H)^147^. Therefore, this mode of virus transmission indicates that the respiratory mucosae explicitly act as the immune system's frontline in response to viruses. Consequently, mucosal immunity has the potential to provide a robust defense to prevent initial infection and subsequent transmission. This represents a significant opportunity for the development of inhaled mRNA vaccines.

#### 5.1.2 Case studies

Mucosal immunization could stimulate mucosal IgA antibodies, which can capture and neutralize respiratory pathogens on the mucosal surfaces, thus providing the first line of defense against infection^156^, ^157^, ^158^. Recent scientific studies have utilized animal models and clinical trials to investigate the efficacy of mucosal vaccines against SARS-CoV-2. These studies have indicated that the combination of mucosal and systemic immunity can provide comprehensive protection against SARS-CoV-2^155^^,^^154^. For example, CanSino Biologics Inc. and Feng et al.^159^ for the first time, reported the safety, mucosal and systematic immunogenicity of inhaled adenovirus type-5 vector-based COVID-19 vaccine employing inhalation that was firstly utilized for SARS-CoV-2 vaccine delivery. The first inhaled COVID-19 vaccine induced robust humoral, cellular, and mucosal immunization responses, including IgG and IgA, and were elicited in all vaccinated people. Meanwhile, Ye et al.^160^ report a vaccine formulation that cannot only induce respiratory mucosal immunity after local lung delivery but is also inhaled as a dry powder, avoiding the need for cold chains and the use of needles. This confirmed that the developed inhalation delivery strategy was to have shots deliverable through the airways to generate mucosal immunity that today's injectable vaccines cannot provide.

**Figure 8:**
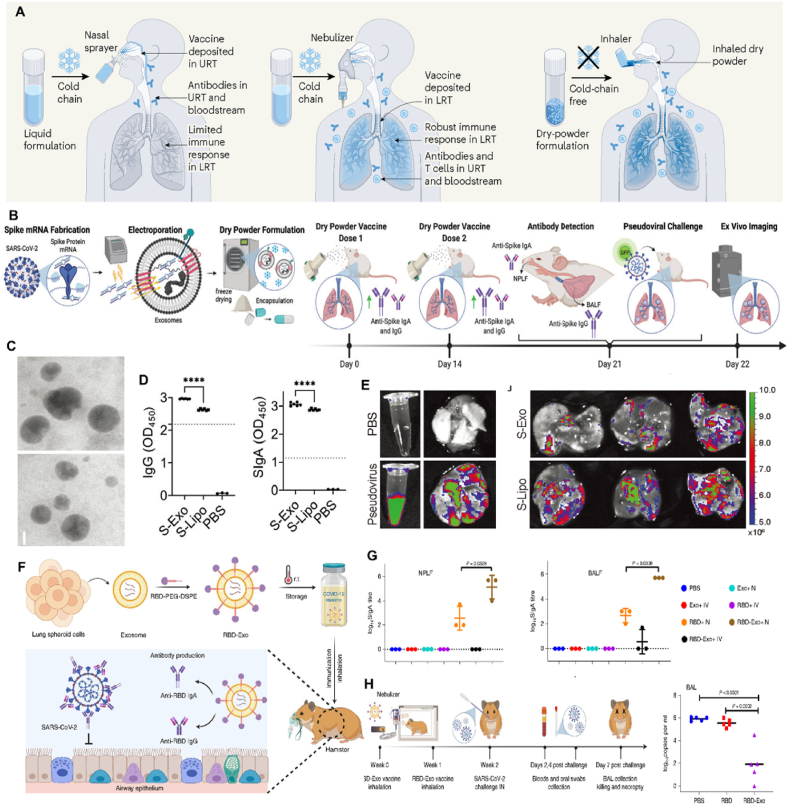
(A) Current injected COVID-19 vaccines didn't generate immunity in mucosal tissues that line the airways at the site of viral entry. Next-generation inhaled vaccines are being developed to boost such immune responses. Reprinted with the permission from Ref. 162. Copyright © 2023 Spring Nature. (B) Schematic of S protein mRNA loading into lung-derived exosomes, dry powder formulation, inhaled vaccine delivery doses, antibody production against SARS-CoV-2 spike protein, and pseudoviral challenge. (C) TEM images of S-Exos and S-Lipos at room temperature. (D) ELISA detected an anti-spike IgG antibody titer from murine BALF, and an anti-spike SIgA antibody titer was detected from murine NPLF. (E) *Ex vivo* images of PBS or pseudovirus in solution (left) and in lungs 24 h after dry powder inhalation (right), and *Ex vivo* images of S-Exo- or S-Lipo-vaccinated (right) lungs 24 h after pseudoviral challenge. Reprinted with the permission from Ref. 55. Copyright © 2022 Elsevier. (F) Schematic representation of the fabrication of the RBD-Exo vaccine, which was delivered into the lungs *via* inhalation. RBD-Exo induced mucosal and systemic immunity by generating RBD-specific IgA and IgG antibodies against SARS-CoV-2 infection in hamsters. (G) ELIS detected RBD-specific SIgA antibody titers from NPLF (left) and BALF (right). (H) Protective effect of the RBD-Exo vaccine in the Syrian hamster model of SARS-CoV-2 infection. Reprinted with the permission from Ref. 163. Copyright © 2022 Spring Nature.

Pulmonary drug delivery can be achieved *via* three different routes of administration: intranasal, intratracheal, and inhalation. Although inhaled vaccines can be delivered nasally^160^, can spread to part of the brain (the olfactory bulb) and are limited mainly to the URT rather than the LRT, thus posing a potential safety concern and efficacy if used in humans. In the second case, intratracheal delivery is an invasive procedure unsuitable for the pulmonary delivery of therapeutics in humans. Inhalation is the most common route and relies on normal breathing to administer aerosolized therapeutics through the airway. Liquid or dry powder vaccines can be inhaled through the mouth using an aerosol-generating nebulizer. The inhalation of the vaccine to the LRT aims to induce a range of immune responses, including the production of antibodies, activation of T cells, and stimulation of the innate immune system. Simultaneously, the vaccine also maintains immune responses in the URT and bloodstream (Fig. 8A). The carriers of actives in inhaled vaccines remain difficult with optimized physiochemical parameters. Most COVID-19 vaccines under clinical development are based on a viral-vector system and are delivered through the nose. Except for inhaled adenoviral delivery systems, exosomes are a class of naturally derived extracellular vesicles (approximately 100 nm in diameter) secreted from most cells. These have raised remarkable attention in drug delivery and immunization areas. Recently, Wang et al.^161^ developed room-temperature-stable inhalable lung-derived extracellular vesicles or exosomes (Lung-Exos) as mRNA and protein drug carriers. Compared with standard synthetic nanoparticle Lipos, Lung-Exos exhibited superior distribution to the bronchioles and parenchyma. They were deliverable to the lungs of rodents and nonhuman primates by dry powder inhalation. In a vaccine application, the SARS-CoV-2 spike (S) protein-encoding mRNA-loaded Lung-Exos (S-Exos) elicited greater IgG and secretory IgA responses than its loaded liposome (S-Lipo) counterpart. Importantly, S-Exos remained functional at room-temperature storage for one month. Their results suggested that extracellular vesicles could serve as an inhaled mRNA drug-delivery system superior to synthetic liposomes (Fig. 8B–E).

Meanwhile, Cheng and coworkers also designed a novel inhalable COVID-19 vaccine based on exosomes, with effective mucosal immune stimulation and long-term stability. Such vaccine consisted of a recombinant SARS-CoV-2 RBD conjugated to lung-derived exosomes, which, concerning liposomes, created the virus-like particles imitating the morphology of authentic virus and enhanced the retention of the RBD in both the mucus-lined respiratory airway and in lung parenchyma (Fig. 8F). In mice, the inhalation vaccine of RBD-Exo VLPs produced the highest amount of SIgA antibodies in nasopharyngeal lavage fluid (NPLF) and bronchoalveolar lavage fluid (BALF) (Fig. 8G). Furthermore, researchers estimated the effect of inhalable RBD-Exo VLPs on preventing high-dose live SARS-CoV-2 infection in the hamster model that could replicate serious diseases in the clinic. Compared with others, RBD-Exo VLPs groups showed lower virus detection amounts and the highest RBD-specific serum antibody concentration in every tested timepoint (Fig. 8H). Therefore, the inhalation vaccine was considered the most promising of all kinds of vaccine delivery strategies. It was expected to improve the people's willingness to vaccinate and further achieve global COVID-19 vaccine popularization. Their study provided a direction for the development of COVID-19 therapy and a reference for the treatment of other respiratory illnesses^161^.

### 5.2 Influenza

**Figure 9:**
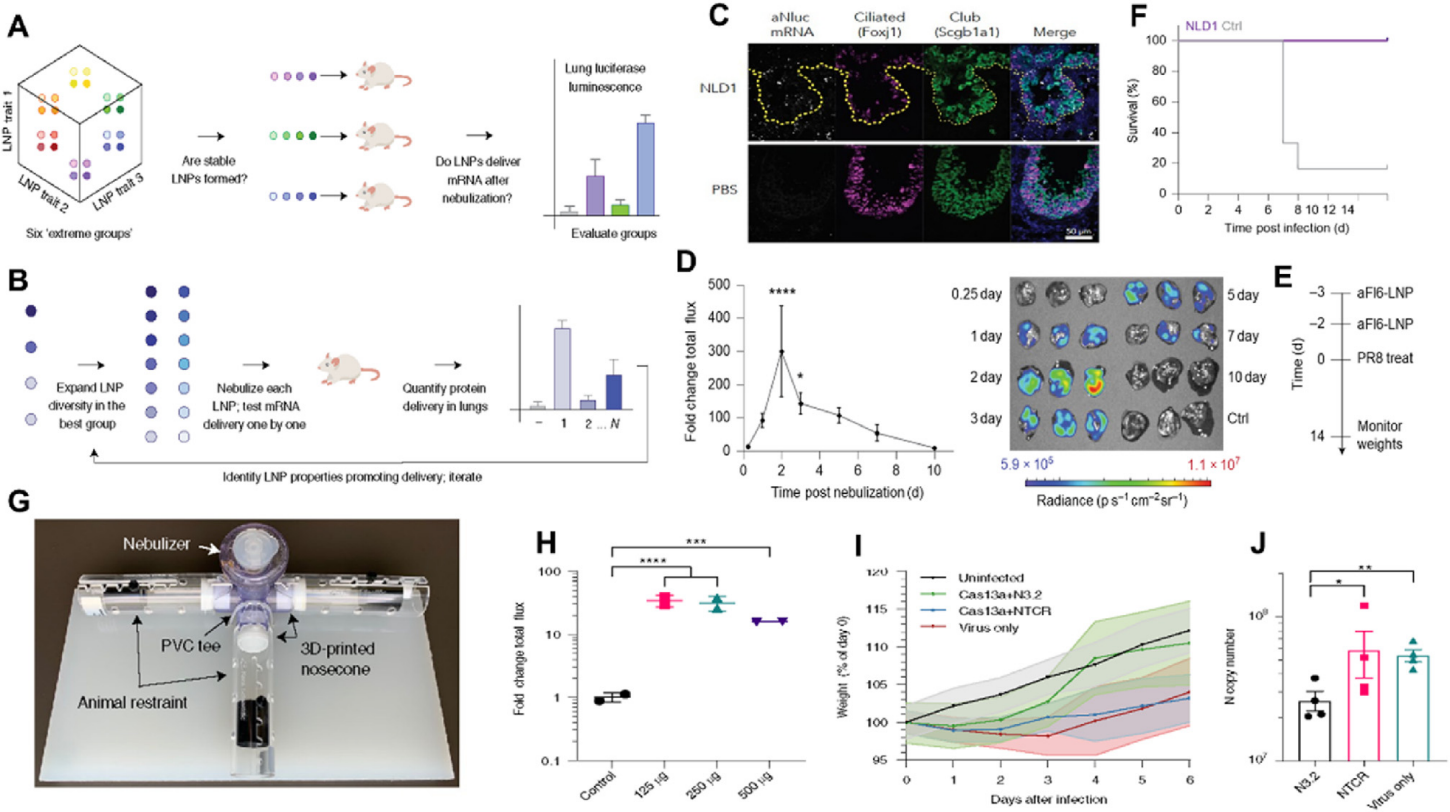
(A, B) An *in vivo* workflow to evaluate how chemically diverse LNPs deliver mRNA to the lung after inhalation. PB2 targeted guide selection and influenza A sequence coverage. (C) RNA-fluorescence *in situ* hybridization analysis for AncNanoLuc mRNA uptake in epithelial cell subtypes. (D) NLD1 carrying AncNanoLuc was administered at a dose of 20 μg per mouse. Protein expression was quantified and imaged over several days. (E) NLD1 treatment regimen for H1N1 study. (F) Survival curves of mice receiving the NLD1 treatments. Reprinted with the permission from Ref. 131. Copyright © 2021 Spring Nature. (G) Inhalable antiviral Cas13a mRNA in rodent and apparatus for mouse studies. (H) Quantitative Analysis of the total flux. (I) Hamsters were dosed with Cas13a mRNA with guides 20 h before infection with SARS-CoV-2. (J) Lung viral loads from hamsters on Day 6 after infection. Reprinted with the permission from Ref. 170. Copyright © 2021 Spring Nature.

Influenza virus is classified into four types: A, B, C, and D. Influenza virus can cause human influenza, avian influenza, swine influenza, equine influenza, and other diseases in humans and animals^164^, ^165^, ^166^. Although more than 130 influenza A subtype combinations have been detected in nature, mainly from wild birds, there are potentially many more influenza A subtype combinations due to the virus's “reassortment” ability^167^. As the virus has zoonotic potential and is capable of an antigenic shift, the gene reassortment of influenza A viruses of different origins results in highly contagious hybrid strains resistant to the existing therapy. Vaccination is the most common prevention strategy for influenza A virus infections. As mentioned in this review, inhaled vaccines stimulate mucosal immunity primarily, effectively preventing pathogen invasion and enhancing the immunoprophylactic effect of vaccination. Therefore, several studies have been conducted on inhalation vaccines for influenza virus prevention and control. Lokugamage et al.^34^ designed an LNP-mRNA named inhaled NLD1 to immunize mice against the influenza virus by optimizing the LNP formulation. They optimized the composition, molar ratios, and structure of LNPs made of lipids, neutral or cationic helper lipids, and PEG by using cluster-based workflows and then allowed for maximizing *in vivo* screening and delivery efficiency (Fig. 9A and B). It was also demonstrated that the mRNA carried by NLD1 could be transferred to ciliated bronchial epithelial cell, bronchial club cells, and alveolar type I and II in the lung epithelial cells and could continue to be translated in the body for up to a week (Fig. 9C and D). As expected with the inhaled viral dose, five of the six control mice died after progressively losing weight. In contrast, all of the mice treated with NLD1 survived, suggesting that this cluster-based screening system provided a novel approach for rapidly treating high-risk infectious diseases (Fig. 9E and F)^34^. Many studies have focused on applying the CRISPR/Cas13a system, which could target RNA for gene cutting.^168^, ^169^ Compared with traditional RNAi technology, Cas13A-mediated gene silencing has advantages, such as high efficiency and low off-targeting rate, thus theoretically having higher safety in disease treatment. Compared with Cas9-mediated gene knockout technology, Cas13D-mediated gene silencing does not alter genomic DNA, indicating that this gene silencing is reversible. Treating some acquired diseases, such as acquired metabolic diseases, is more advantageous. Santangelo et al. designed CRISPR RNAs specific for PB1 and highly conserved regions of PB2 of the influenza virus and selected the crRNAs that reduced viral RNA levels most efficiently in cell culture. A nebulizer delivered the polymer-formulated Cas13a mRNA and the validated guides to the respiratory tract. In mice, Cas13a degraded influenza RNA in lung tissue efficiently after inhalation. This finding suggested that Cas13a-mediated targeting of pathogenic viruses could mitigate respiratory infections (Fig. 9G–J)^48^. In general, regarding influenza virus prevention and control, most studies mainly focus on optimizing nucleic acid delivery vectors and screening targets. Although this 7C1 vector based on PEI improved the inhaled delivery efficiency of LNP through prescription optimization, only a few vectors have been reported so far. Therefore, further research on nucleic acid inhalation carriers is imperative to provide insights for future efficiency development. At the same time, further exploration of the broad spectrum of ways to combat influenza viruses should be conducted, and new technologies and new antiviral targets should also be discovered.

### 5.3 Idiopathic pulmonary fibrosis (IPF)

**Figure 10:**
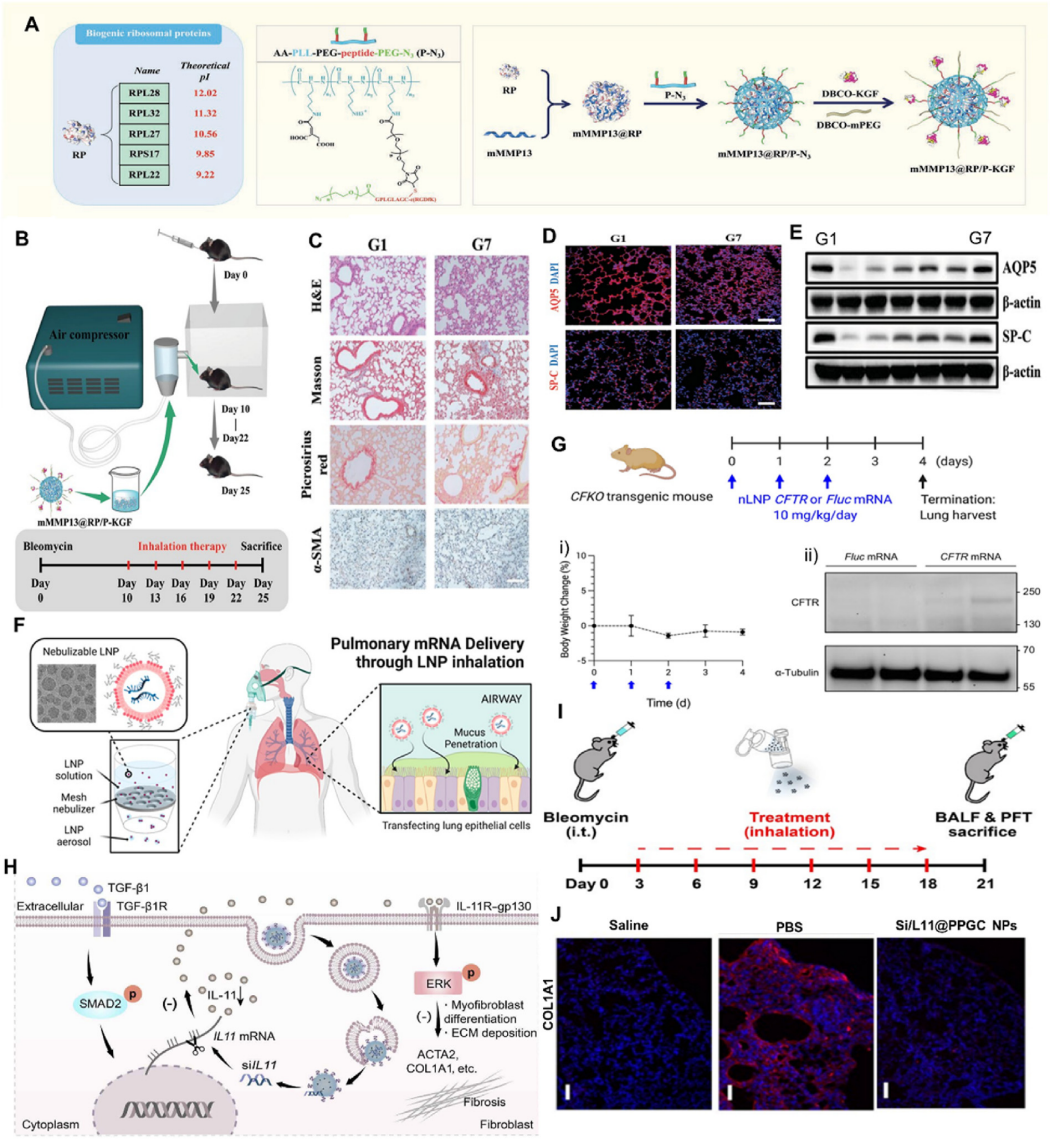
(A) A class of biogenic ribosomal proteins with various theoretical pls and the construction of mMMP13@RP/P-KGF with MMP2-responsive and pH-sensitive abilities. (B) Schematic of the study design. (C) Representative histologic analyses of lung sections. (D,E) Representative immunostaining and Western blot assay of lung sections for determining surfactant protein C and aquaporin 5. Reprinted with the permission from Ref. 50,106. Copyright © 2022 John Wiley and Sons. (F) Inhaled LNP deliver mRNA encoding CFTR for the treatment of IPF. (G) A dosing regimen for CFTR mRNA delivery *via* inhalation. (i) Body weight change of CFKO transgenic mice after repeat dosing (blue arrow). (ii) Western blot images after immunoprecipitation using an anti-CFTR antibody. mRNA delivered by inhalation is noted above the images. Upper and lower blots were probed using anti-CFTR and anti-*α*-tubulin antibodies, respectively. Reprinted with the permission from Ref. 38. Copyright © 2022 American Chemical Society. (H) Inhaled delivery of siRNA-encapsulated PPGC NPs to MLFs for treating IPF. (I) Experimental design of the animal study. (J) Representative immunofluorescence images of COL1A1 from mouse lung sections treated with indicated treatment. Reprinted with the permission from Ref. 75. Copyright © 2022 The American Association for the Advancement of Science.

IPF, a lethal respiratory disease with few treatment options, occurs due to repetitive micro-injuries to alveolar epithelial cells and progresses with an overwhelming deposition of extracellular matrix, ultimately resulting in fibrotic scars and destroying the alveolar architecture. IPF can cause lung infection, respiratory failure, pulmonary hypertension, pulmonary heart disease, heart failure, and other symptoms. Nowadays, inhaled delivery of nucleic acid drugs to treat IPF has been reported in many cases. Zhang et al.^50^ constructed a nanoparticle assembled by a ribosomal protein-condensed mRNA core, a bifunctional peptide-modified corona, and a keratinocyte growth factor (KGF) with a PEGylated shielding shell. When inhaled *via* a nebulizer, the nanoformulations carried by microdrops were deposited in the alveoli and penetrated fibrotic foci, where the outer KGFs were detached after matrix metalloproteinase 2 triggering. The core of the RGD-based graft then exposed and specifically targeted cells with elevated integrins for intracellular delivery of mRNA. The results suggested that repeated nanomaterial inhalation could synergically improve bleomycin-induced lung function in mouse models by promoting intralesional expression of MMP13 and KGFS-mediated alveolar re-epithelialization to accelerate local collagen clearance. In addition, Sahay et al. constructed an LNP based on ionizable lipids of DLin-MC3-DMA and substituted *β*-sitosterol for cholesterol to deliver mRNA encoding the cystic fibrosis transmembrane conductivity regulator (CFTR). The formulation was delivered to an animal model of CFTR deficiency after inhalation, resulting in lung expression of this therapeutic protein, significantly improving IPF (Fig. 10A–E)^50^. Kim et al.^38^ utilized PEG lipids to enhance the surficial stability of LNPs by including a cholesterol analogue, *β*-sitosterol, to improve endosomal escape. Increased PEG concentrations in LNPs enhanced the shear resistance and mucus penetration, while *β*-sitosterol provided LNPs with a polyhedral shape, facilitating endosomal escape. This study demonstrated the rational design approach for cystic fibrosis of inhalable LNP-based mRNA therapies (Fig. 10F and G).

Furthermore, Bai et al.^75^ obtained siIL11@PPGC NPs using nanoparticles prepared by lipid compounds coated with siRNA for IL11, which has the following outstanding advantages: 1) It has good stability, can withstand the shear force generated in the process of inhalation and keeps the particle size, morphology, cell uptake and encapsulation rate unchanged before and after inhalation; 2) could be condensed into a small volume; 3) After inhalation, it passed through the lung mucus layer and reached deep into the lung tissue. In vitro experiments showed that siIL11@PPGC NPs could significantly inhibit fibroblast conversion to myofibroblast, extracellular matrix deposition, and migration. In bleomycin-induced pulmonary fibrosis in mice, aerosol inhalation of siIL11@PPGC NPs effectively reduced the expressions of ACTA2 and COL1A1, significantly reduced hydroxyproline content, and improved fibrosis area and collagen content (Fig. 10H–J). The inhaled RNA drugs have a significant anti-fibrosis effect and significantly improve lung function in mice with pulmonary fibrosis, providing a new therapeutic method for repairing lung tissue damage and intervening in functional recovery after pulmonary fibrosis.

### 5.4 Asthma

**Figure 11:**
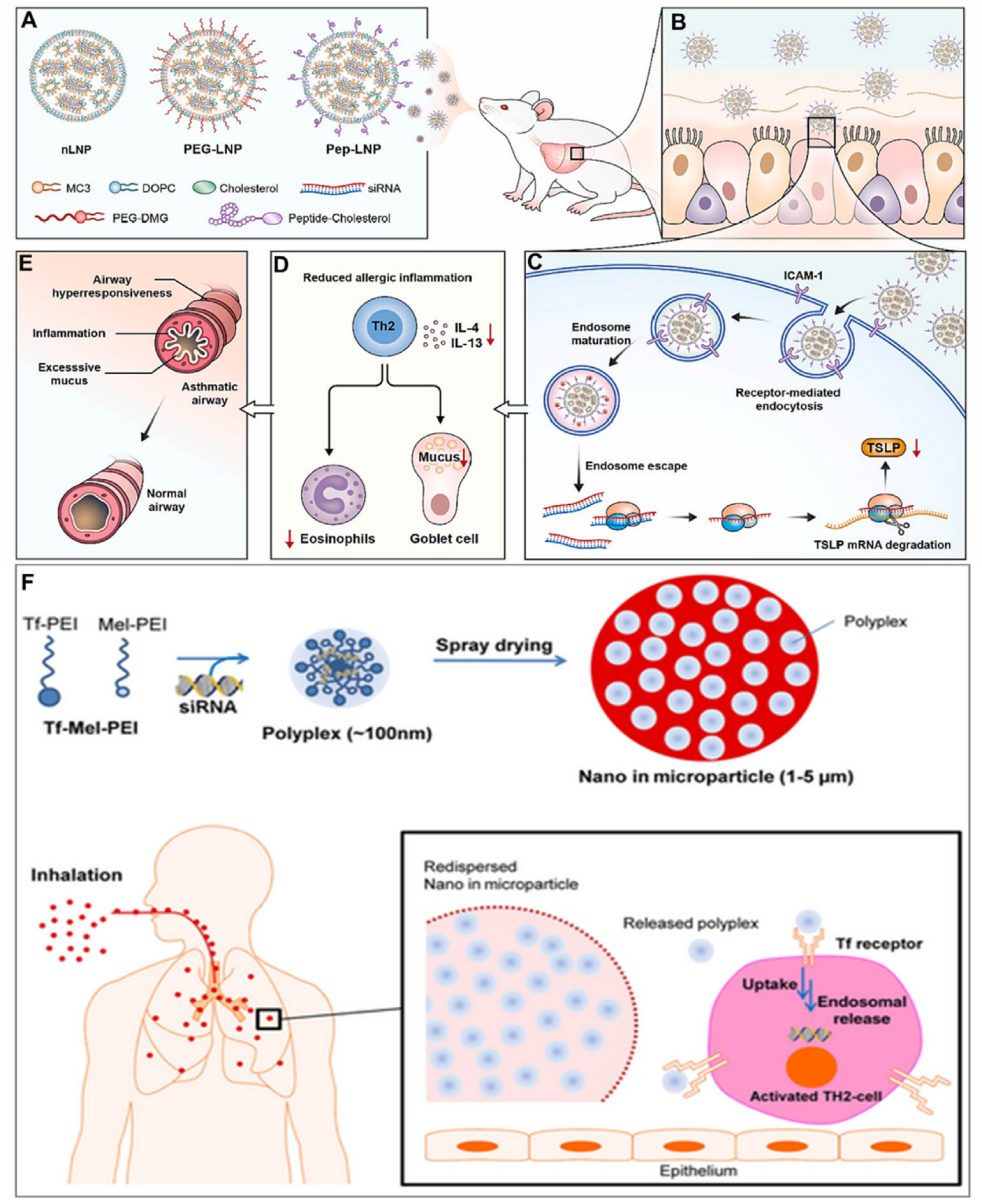
(A) Schematic illustration of AECs-specific delivery of LNP-siTSLP alle*via*ted allergic asthma *via* pulmonary administration. (B) Deposition of LNP-siRNA in the airway post pulmonary administration. (C) ICAM-1 receptor-mediated endocytosis of Pep-LNP-siTSLP by AECs and the following RNA interfering action. (D) Suppression of the production of T helper 2 cytokines, eosinophils infiltration and mucin over-secretion. (E) Alleviated airway inflammation in allergic asthma. Reprinted with permission from Ref. 174. Copyright © 2022 Elsevier. (F) Polyplexes made from siRNA complexed with Tf-PEI and Mel-PEI were shown to be specifically taken up by ATCs, facilitating the endosomal release necessary for efficient gene silencing. Dry powders were obtained for pulmonary delivery as a therapeutic option in asthma treatment by spray drying. Reprinted with the permission from Ref. 175. Copyright © 2022 Wiley Periodicals, Inc.

Asthma is a chronic, noncommunicable disease characterized by various degrees of airway inflammation, obstruction, mucus hypersecretion, and hyperresponsiveness. It represents the most prevalent and intractable disease due to its complex pathophysiology and multifactorial aetiology^163^. The global prevalence of asthma in adults is 4.3% and causes death in approximately 0.4 million people annually worldwide^162^^,^^171^. Traditional treatment methods, such as use of bronchodilators, anti-inflammatory drugs and Reducing mucolytic agent, could provide only symptomatic relief but hardly prevent the progressive deterioration of lung function in asthmatic patients^172^. It has recently been reported that inhaled delivery systems are fruitful in treating asthma^37^^,^^173^. For example, Zhang et al.^37^ exploited novel inhaled LNP targeting intercellular adhesion molecule-1 (ICAM-1) receptors on the apical side of AECs. A cyclic peptide that resembled part of the capsid protein of rhinovirus and bound to ICAM-1 receptor was initially conjugated with cholesterol and subsequently assembled with ionizable cationic lipids to form the LNP (Pep-LNP) loaded with siRNA against thymic stromal lymphopoietin (TSLP siRNA). After inhalation of the Pep-LNP-siTSLP, it was engulfed by AECs by ICAM-1 receptor-mediated endocytosis and remarkably downregulated the expression of TSLP in AECs and effectively alleviated inflammatory cell infiltration, and reduced the secretion of other proinflammatory cytokines (Fig. 11A–E). These results suggested that inhaled Pep-LNP-siTSLP could be a promising therapy to alleviate epithelium-mediated inflammatory responses in asthmatic conditions. Another recent study confirmed the feasibility of efficient inhaled lung drug delivery for asthma treatment. Keil et al.^170^ first identified a suitable transferrin receptor-mediated uptake pathway to target efficiently and specifically activated TH2 cells with a melittin-PEI conjugate, forming polyplexes with siRNA. The new formulation showed improved endosomal escape and gene silencing efficacy. Additionally, to develop a clinically relevant dosage form for pulmonary delivery of siRNA, they have lately focused on a dry powder formulation by spray drying to produce inhalable nano-in-microparticles. Their efforts were devoted to the development of a novel treatment for asthma that could be translated from bench to bedside. In this work, the inhalation of multifunctional nanogels was developed for asthma treatment (Fig. 11F). Therefore, the application of inhaled RNA therapy in asthma disease will provide new ideas for the treatment of asthma and other related diseases.

### 5.5 Lung cancer

**Figure 12:**
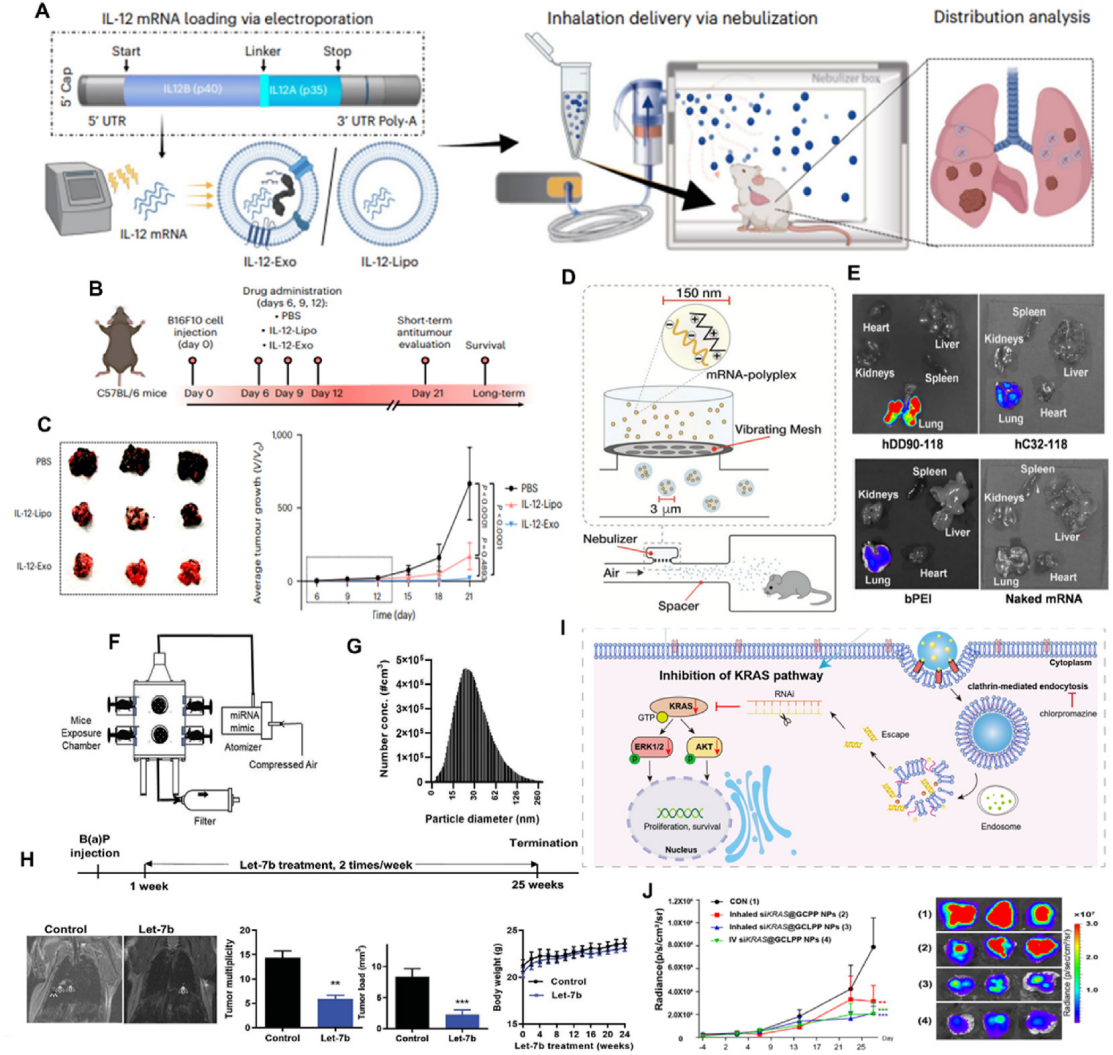
(A) Schematic showing IL-12 mRNA loading into HEK-Exo (IL-12-Exo) or liposomes (IL-12-Lipo), followed by nebulized inhalation administration to LL/2 tumor-bearing mouse lungs. (B) Schematic showing the establishment of the B16F10 melanoma lung metastatic tumor model and anti-metastatic tumor assessments after mice were administered with different treatments. (C) Representative lung morphologies 21 days after B16F10 tumor inoculation. Black points indicate the metastatic foci. Reprinted with the permission from Ref. 53. Copyright © 2024, Springer Nature. (D) A vibrating mesh nebulizer connected to a whole-body chamber delivered IVT-mRNA encoding for firefly luciferase to mice. (E) bioluminescence in the lung 24 h after inhaled delivery of mRNA. Reprinted with the permission from Ref. 8. Copyright © 2019 John Wiley and Sons. (F) Schematic diagram of the aerosol delivery system. (G) Size distribution of let-7b miRNA mimic particles in the exposure chamber. (H) Efficacy of inhaled let-7b miRNA in the B(a)P-induced lung cancer model. Reprinted with the permission from Ref. 56. Copyright © 2021 John Wiley and Sons. (I) The design of inhaled siKRAS@GCLPP NPs for KRAS-mutant non-small-cell lung cancer NPs includes. (J) Quantitative BLI light intensity of the chest before and after the treatment. Reprinted with the permission from Ref. 45. Copyright © 2022 American Chemical Society.

Lung carcinoma is one of the most common cancers and has one of the lowest survival rates in the world. RNA-based therapies have gained much attention as biomedicines due to their remarkable therapeutic effects and high specificity and potency. Inhaled RNA drugs for the treatment of lung disease may have a good potential for application. This chapter summarized the use of inhaled delivery systems for different types of RNA in anti-tumor research for lung cancer. Liu's group^53^ developed an inhalable exosome loaded with IL-12 mRNA for inhaled mRNA therapy. Inhalation administration leads to targeted administration and fewer systemic side effects, with IL-12 mRNA producing interferon-gamma in both the primary and adaptive immune cell populations. Activation of the tumor microenvironment increases immunogenicity, resulting in a more robust immune response. This enhanced response facilitates the expansion of cytotoxic immune cells, immune memory formation, improved antigen presentation, and activation of tumor-specific T cells. This approach shows promise in treating both *situ* and metastatic lung tumors (Fig. 12A–C). Similarly, Patel et al.^8^ synthesized hPBAEs to enable the nanoformulation of stable and concentrated polyplexes suitable for inhalation. This strategy achieves uniform distribution of luciferase mRNA throughout all five lung lobes and produces stable luciferase protein expression 24 h after inhalation of hPBAE polyplexes. Delivery is localized to the lung; no luminescence is observed in other tissues. Furthermore. Repeated dosing of inhaled hPBAE-mRNA generates consistent protein production in the lung without local or systemic toxicity. The results indicate that inhaled delivery of IVT-mRNA facilitated by hPBAE vectors may provide a clinically relevant delivery system for lung cancer (Fig. 12D and E). In the inhaled miRNA study, Zhang et al.^56^ investigated the efficacy of inhaled let-7b miRNA treatment in lung cancer prevention. Inhaled let-7b mimic showed significant inhibition of lung adenoma by immune-promoting effects *via* downregulating PD-L1 in tumors or PD-1 on CD8^+^ T cells. These changes potentiated antitumor CD8^+^ T cell immune responses. The results suggested that this inhaled let-7b mimic was a promising approach for lung cancer prevention (Fig. 12F–H). Finally, research on the inhaled delivery of siRNA for lung cancer has also demonstrated its tremendous potential. Zhao et al.^45^ investigated the antitumor efficacy of inhaled siKRAS@GCLPP NPs for KRAS-mutant non-small-cell lung cancer utilizing a murine orthotopic lung cancer model. Their findings suggested that inhaled siKRAS@GCLPP NPs could deliver equivalent effectiveness to intravenously injected NPs while reducing the adverse effects associated with systemic administration. Inhalable siKRAS@GCLPP NPs showed significant tumor-targeting capability and enhanced antitumor activity in an orthotopic mouse model of human KRAS-mutant NSCLC. This work presented a new avenue for noninvasive inhaled siRNA delivery that holed tremendous potential for treating KRAS-mutant NSCL (Fig. 12I and J). The above studies indicated that RNA delivered through the respiratory tract had good research value for lung cancer treatment. However, there are still few relevant studies and more attempts are needed to develop new delivery systems, select new targets, and explore more clinical translational approaches.

### 5.6 Obstructive pulmonary disease (COPD) and tuberculosis (TB)

**Figure 13:**
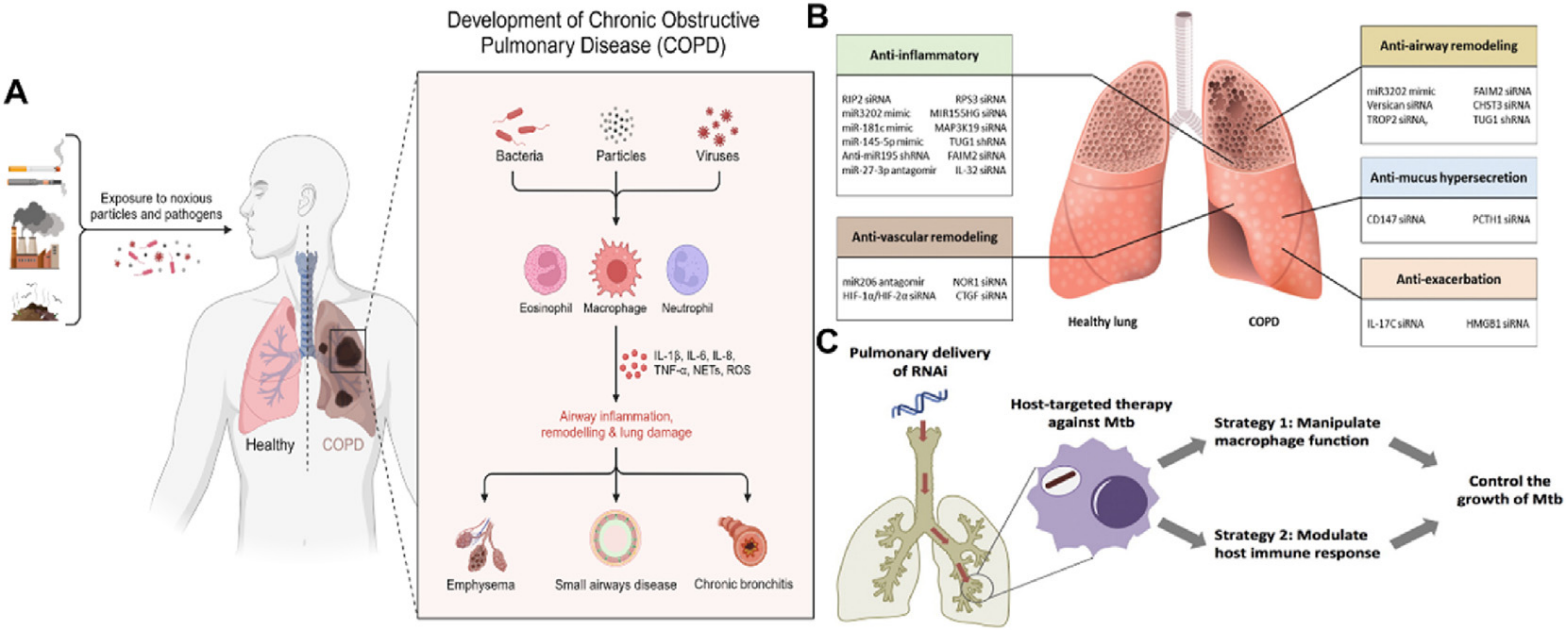
(A) Chronic obstructive pulmonary disease (COPD) results in airway inflammation, remodelling, and lung damage. Created with BioRender.com (B) Pharmacological actions of noncoding RNA molecules as potential therapeutics for COPD. Reprinted with the permission from Ref. 181. Copyright © 2020 Elsevier Science London. (C) The inhaled delivery of RNAi molecules as therapeutics against TB. Reprinted with the permission from Ref. 155. Copyright © 2023 Elsevier.

COPD is a common severe chronic pulmonary disease characterized by airway inflammation, airflow obstruction, as well as damage to the lung parenchyma. COPD is a complex and multifactorial respiratory disease that is caused by environmental factors, for example, tobacco smoking, air pollution, allergens, genetic factors, or occupational risks. Major pathological features of COPD are obstructive bronchiolitis, emphysema, pulmonary hypersecretion, and small airway obstruction (Fig. 13A)^176^. Current therapeutics for COPD are largely borrowed from the drug armamentarium for the treatment of asthma, which has different pathophysiological mechanisms from COPD. COPD shows a predominant involvement of peripheral airways (bronchioles) and lung parenchyma, which COPD has been linked to dysregulated expression of mRNAs and noncoding RNAs, including miRNAs, PIWI-interacting RNAs, long noncoding RNAs, and circular RNAs (Fig. 13B)^177^. Identifications of disease-triggering pathways and gene targets for COPD have opened an opportunity for inhaled RNA therapeutics. However, clinical translation has remained limited due to insufficient understanding of the intricate disease mechanism and a lack of robust and predictive animal models. TPI 1100, which consists of ASO targeting phosphodiesterases (PDE4 and PDE7), is the only reported RNAi-based product launched into phase I clinical trial to treat COPD. However, the pipeline was soon terminated. Recent pre-clinical experiments have used non-RNA drug inhalation therapy^178^ or RNA drug pulmonary administration (non-inhalation therapy)^179^ and have been mainly dedicated to targeting inflammatory cytokines. Of note, more and more research on inhaled RNA therapy may shift the current paradigm of COPD management. Tuberculosis is the infectious disease that causes the most deaths per year worldwide. TB is caused by Mycobacterium tuberculosis (Mtb), which most frequently enters the body through the respiratory route. Despite the potential therapeutic regimens comprising both the conventional drugs (isoniazid and rifampicin) and the newly developed chemotherapy approaches that are now undergoing various stages of clinical trials, the threatening prevalence of multidrug-resistant and extensive-drug resistant-TB keeps calling for safer and more efficient treatment methods. Moreover, exposure of the mycobacteria to sub-therapeutic levels of anti-TB drugs during treatment is a driver for the emergence of drug-resistant microbial strains^180^. One effective approach to increase the bactericidal effect of antibiotics is to achieve a high local concentration of the drug by localized drug delivery, such as inhalation therapy. Although the COVID-19 pandemic has impacted TB diagnosis and treatment caused by the SARS-CoV-2, the RNA-based may present a golden opportunity to accelerate the development and approval of new potentially effective TB vaccine candidates. RNA interference (RNAi) therapy targeting TB is being investigated to enhance host antibacterial capacity or improve drug efficacy on drug resistance strains while minimizing the associated adverse effects. One of the key challenges of RNAi therapeutics arises from the delivery of the RNAi molecules into the target cells. Inhalation could be a direct administration route for treating pulmonary TB non-invasively (Fig. 13C)^174^. The clinical pipeline includes an mRNA-based candidate (BNT164a1/BNT164b1, BioNTech) recently entering phase I clinical trials in Germany (NCT05547464) and South Africa^175^. Recently, this pioneering project, “RNA inhaled vaccines against Tuberculosis”, led by Certest Biotec in collaboration with the University of Zaragoza and the University of the Basque Country, aims to develop a new inhaled RNA vaccine against tuberculosis as a booster for current and future vaccines against the disease. All of the above studies indicate the potential of inhaled RNA therapy in treating obstructive pulmonary disease and tuberculosis.

## 6 Conclusions and perspective

In the past decade, RNA therapies have had an exciting potential to greatly improve our understanding of disease and treatment. Inhalation delivery of therapeutic aerosols in treating respiratory tract diseases allows for targeted delivery, minimizing off-target dosing and associated side effects. This review systematically expounds on the principle, RNA classifications, and application of inhalation delivery systems. We first mentioned the nebulizers used in the inhalation systems and compared the characteristics of different inhalers. Different nebulizers have their advantages and disadvantages, and they have a diverse application range. Of these devices, nebulizers and DPIs are of particular interest since they are distributed easily and the administration is simple. In terms of inhaled vaccines, we hope it has been deposited in the lower respiratory tract. Liquid or dry powder vaccines can also be inhaled through the mouth using an aerosol-generating nebulizer or dry powder inhaler. Meanwhile, we hope to make nebulizers more portable and simplified, which could greatly optimize patient use. Different patients need to deposit the drug in different places, and the smaller the particle, the deeper the drug can go into the respiratory tract. Moderate inhalation power is suitable for ordinary use, but for acute attack patients, it is more suitable for immediate high-power inhaled treatment. Therefore, the power of inhalers and aerosol particle size can also become controllable, which will become the development trend That is convenient for carrying and cleaning. In addition, Early method development work can ascertain the maintenance of desired integrity for a gene therapy formulation–device combination. For particularly sensitive formulations, optimization of nebulizers and other delivery devices is crucial to mitigate aerosolization stresses in inhalation therapy. Despite promising preclinical findings on candidate inhaled RNA therapies, the outcomes of human trials have been disappointing. The reason is that the challenges encountered in inhalation therapy pertain to numerous biological and otherwise barriers that impede the effective treatment of diseases through RNA therapy aerosols. The distribution of inhaled therapeutics in the lungs is determined by the aerodynamic diameter of RNA loaded. Such complex anatomical patterns will further increase the barriers to the clinical translation of RNA therapeutics to human patients. The other reason lies in using mice as the animal model in most *in vivo* investigations. Due to major differences in lung physio-anatomy between mice and humans, the pre-clinical data cannot be easily extrapolated to human patients. We therefore recommend using lung on a chip, *in vitro* cell models, larger animal models (*e.g.*, non-human primates, ferrets and pigs), and developing more inhalation-based RNAi therapeutics to increase the probability of future use in clinics. Next, we summarized the research progress on siRNA, ASO, and other types of RNA inhaled delivery. A related advance was the need to understand how chemical modifications to the RNA payload influence RNA stability avoidance of intracellular off-target effects. Much of the clinical and preclinical research has focused on siRNA and mRNA for the inhaled delivery of RNA. With the vast growth of RNA-based therapies, Various other types and functions of RNA will also be attempted for inhalation delivery to treat respiratory infections and other pulmonary diseases.

To better accelerate the clinical translation of non-viral platforms, rigorous material screening, and optimization should be conducted to maximize RNA delivery efficiency. Although various types of inhaled delivery carriers have been reported, such as chemical delivery carriers, including polymer, polycomplex, cationic lipid, and ionizable lipids, these delivery systems all have their specific advantages. Polymer and polycomplex have the advantages of stable delivery and high efficiency, but quality control is difficult. However, the traditional cationic lipid has the advantages of a high encapsulation rate and stable transfection, but its safety and delivery efficiency are not as good as ionizing lipids. Currently, two commercially available mRNA vaccines are based on ionizable lipid delivery systems, which seem to have obvious advantages. However, more studies are still needed on optimizing lipid structure and delivery system prescription. Recently, with the rapid development of more ionizable lipid materials and formulation optimization of LNPs, such as CKK-E12, DLin-MC3-DMA, G0-C14, SM102, IR-117-17, IR-19-Py and C12-200, the field of nebulized mRNA delivery have attracted increasing attention. Additional research should be pursued, and more innovations should be made in the structure of ionizable lipids and prescription optimization of delivery systems. Another kind of biomaterial delivery system is also used for inhaled delivery of RNA. Such carriers include AAV and EVs. Although these biological delivery carriers can successfully deliver nucleic acid drugs to the lungs, it is still unclear which biological delivery system is suitable for inhaled delivery of nucleic acid drugs—inhaled delivery carriers, whether viral or nonviral vectors have advantages and disadvantages. In addition, few studies have compared these biomaterial delivery vectors with chemical delivery vectors. Therefore, we are not sure which type of delivery nonviral vectors are more suitable for inhaled delivery of nucleic acid drugs, and much relevant research is still needed.

RNA inhalation delivery can be used to treat various diseases, and it is reported in SARS-CoV-2, Influenza, pulmonary fibrosis, Asthma, lung cancer, and other pulmonary diseases. Future research should focus on an efficient and safe delivery system, selecting potential disease targets for RNA therapies, designing specific drugs for specific viral infections, and broad-spectrum antiviral drugs for multiple viral infections to address the challenges of viral mutations. In cancer biology, the researchers found that using genetically engineered mice more accurately recreated clinical results from human trials carried out in parallel. Suppose we can expand our understanding of how the RNA drug, drug delivery system, and body interact. In that case, patients will benefit from effective next-generation inhaled RNA therapies. Regrettably, preclinical research for inhalation RNA delivery remains somewhat under-described in the literature, perhaps due to the nascent nature of the field. We encourage researchers and sponsors to publish methods and techniques used during preclinical evaluations of inhalation RNA therapy products to aid in refining experimental setups and analysis for future inhaled RNA therapy programs. We expect to see accelerating interest in gene therapy products targeting inhalation delivery. We may seek application innovations in RNA inhalation delivery to develop atomized drugs suitable for various diseases in the coming years.

## Author contributions

Cheng Huang: Writing – original draft, Conceptualization. Hongjian Li: Writing – original draft. Xing Duan: Writing – original draft. Peidong Zhang: Writing – original draft. Shaolong Qi: Writing – original draft. Jianshi Du: Writing – review & editing. Xiangrong Song: Writing – review & editing. Aiping Tong: Writing – review & editing. Guocan Yu: Writing – review & editing, Supervision, Funding acquisition, Conceptualization.

## Conflicts of interest

The authors have no conflicts of interest to declare.
